# Rapid tissue prototyping with micro-organospheres

**DOI:** 10.1016/j.stemcr.2022.07.016

**Published:** 2022-08-18

**Authors:** Zhaohui Wang, Matteo Boretto, Rosemary Millen, Naveen Natesh, Elena S. Reckzeh, Carolyn Hsu, Marcos Negrete, Haipei Yao, William Quayle, Brook E. Heaton, Alfred T. Harding, Shree Bose, Else Driehuis, Joep Beumer, Grecia O. Rivera, Ravian L. van Ineveld, Donald Gex, Jessica DeVilla, Daisong Wang, Jens Puschhof, Maarten H. Geurts, Athena Yeung, Cait Hamele, Amber Smith, Eric Bankaitis, Kun Xiang, Shengli Ding, Daniel Nelson, Daniel Delubac, Anne Rios, Ralph Abi-Hachem, David Jang, Bradley J. Goldstein, Carolyn Glass, Nicholas S. Heaton, David Hsu, Hans Clevers, Xiling Shen

**Affiliations:** 1 Woo Center for Big Data and Precision Health, Pratt School of Engineering, Duke University, Durham, NC, USA; 2 Oncode, Hubrecht Institute, Royal Netherlands Academy of Arts and Sciences (KNAW) and University Medical Center (UMC) Utrecht, Uppsalalaan 8, 3584 CT Utrecht, the Netherlands; 3 Department of Biomedical Engineering, Pratt School of Engineering, Duke University, Durham, NC, USA; 4 College of Arts and Sciences, University of Chapel Hill, Chapel Hill, NC, USA; 5 Biology Department, Trinity School of Arts and Sciences, Duke University, Durham, NC, USA; 6 Xilis, Inc., Durham, NC, USA; 7 Department of Molecular Genetics and Microbiology, School of Medicine, Duke University, Durham, NC, USA; 8 Princess Máxima Center for Pediatric Oncology, Heidelberglaan 25, 3584 Utrecht, the Netherlands; 9 Cancer Genomics Netherlands, Oncode Institute, 3584 Utrecht, the Netherlands; 10 Microbiome and Cancer Division, German Cancer Research Center (DKFZ), Im Neuenheimer Feld 280, 69120 Heidelberg, Germany; 11 Department of Head and Neck Surgery and Communication Sciences, School of Medicine, Duke University, Durham, NC, USA; 12 Department of Pathology, School of Medicine, Duke University, Durham, NC, USA; 13 Division of Medical Oncology, Duke Cancer Institute, Duke University, Durham, NC, USA; 14 Terasaki Institute, Los Angeles, CA, USA

**Keywords:** micro-organospheres, organoid, patient derived organoid, SARS-COV-2, CAR-T, deep learning, cytostatic, cytotoxic, drug resistant, demulsification

## Abstract

*In vitro* tissue models hold great promise for modeling diseases and drug responses. Here, we used emulsion microfluidics to form micro-organospheres (MOSs), which are droplet-encapsulated miniature three-dimensional (3D) tissue models that can be established rapidly from patient tissues or cells. MOSs retain key biological features and responses to chemo-, targeted, and radiation therapies compared with organoids. The small size and large surface-to-volume ratio of MOSs enable various applications including quantitative assessment of nutrient dependence, pathogen-host interaction for anti-viral drug screening, and a rapid potency assay for chimeric antigen receptor (CAR)-T therapy. An automated MOS imaging pipeline combined with machine learning overcomes plating variation, distinguishes tumorspheres from stroma, differentiates cytostatic versus cytotoxic drug effects, and captures resistant clones and heterogeneity in drug response. This pipeline is capable of robust assessments of drug response at individual-tumorsphere resolution and provides a rapid and high-throughput therapeutic profiling platform for precision medicine.

## Highlights

• MOSs provide miniature and scalable 3D tissue models that retain tissue features
• AI imaging MOS assays reliably and rapidly test drug responses on patient biopsies
• MOSs enable direct viral-tissue interaction to screen anti-COVID-19 therapy efficacy
• A MOS T cell potency assay assesses CAR-T cell efficacy against patient tumors

## Teaser

Dr. Wang and colleagues have developed micro-organosphere (MOS) technology that establishes miniature 3D tissue models rapidly in a scalable, high-throughput fashion. The unique features of MOSs enable direct viral infection for COVID-19 drug screens, immune cell penetration for a CAR-T potency assay, and assessment of nutrient dependence. Combined with AI-imaging analysis, MOSs offer a clinically feasible assay to guide precision oncology.

## Introduction

Immortalized cell lines and genetically engineered mice have been workhorses for preclinical functional assays for several decades (Bose et al., 2021). Recently, patient-derived xenograft (PDX) and organoid (PDO) models have been shown to correlate with clinical outcomes (Letai et al., 2022). However, the application of PDX models in clinical settings is limited by their relatively low uptake rate and speed of development, high cost, and limited throughput. Compared with PDXs, PDOs offer higher rates of establishment and throughput and are less time consuming. Studies have shown that PDO responses to chemotherapy or radiation therapy are largely consistent with patient responses and could potentially serve as avatars for therapeutic decision-making (Ganesh et al., 2019; Sachs et al., 2018; Sato et al., 2011; Tiriac et al., 2018; van de Wetering et al., 2015; Vlachogiannis et al., 2018; Yao et al., 2020). Yet, several major hurdles hamper the clinical translation of PDOs: (1) it generally takes weeks to expand cultures from clinical samples to have adequate PDOs for drug testing; (2) for rapid clinical diagnostics to guide cancer treatment, the presence of endogenous stromal cells in passage zero (p0) PDOs can confound bulk cell viability readout; (3) the bulk basement membrane extract (BME) dome is not conducive to efficient penetration by immune cells and virus for studying cell therapies and host-pathogen interactions (Schnalzger et al., 2019); and (4) the highly manual process to grow PDOs depends on individual operators’ skills and is not conducive to reproducibility in clinical settings.

Technologies such as droplet microfluidics and microcavity arrays can be leveraged to increase the speed or throughput of spheroid or organoid-based assays (Brandenberg et al., 2020; Jiang et al., 2020; Tomasi et al., 2020). Several attempts have also been made recently to grow stem cells and minced tissues in hydrogel micro-beads (Allazetta and Lutolf, 2015; Fang et al., 2021; Liu et al., 2020; Schindler et al., 2021; Wang et al., 2020). However, it remains challenging to record reliable readouts from drug assays using p0 cultures derived from fresh tissue due to heterogeneous cell composition and growth patterns. Furthermore, a scalable *in vitro* tissue model platform to enable a broad array of applications including oncology, infectious disease, and nutrient absorption will address critically unmet needs.

We developed micro-organospheres (MOSs) using emulsion microfluidics, which can be established rapidly from patient tumor tissues or PDOs and recapitulate key characteristics (e.g., morphology, polarity, and differentiation) and drug responses. Uniform nutrient uptake in a duodenum MOS model allowed us to assess the effects of different dietary sugars. The small size and large surface-to-volume ratio of MOSs enabled direct viral infection of host epithelium for severe acute respiratory syndrome coronavirus 2 (SARS-CoV-2) drug testing and efficient T cell penetration for a rapid chimeric antigen receptor (CAR)-T potency assay. Furthermore, we established a scalable pipeline for automated MOS seeding, treatment, and imaging. Coupled with a neural-network-based machine-learning algorithm and live/dead labeling strategy, we were able to rapidly assess drug response by overcoming plating variation, differentiating drug cytostatic/cytotoxic effects, capturing drug-resistant clones, distinguishing tumorspheres from stroma, and capturing heterogeneous treatment response at the individual-tumorsphere resolution.

## Results

### Development of the MOS technology

**Figure 1:**
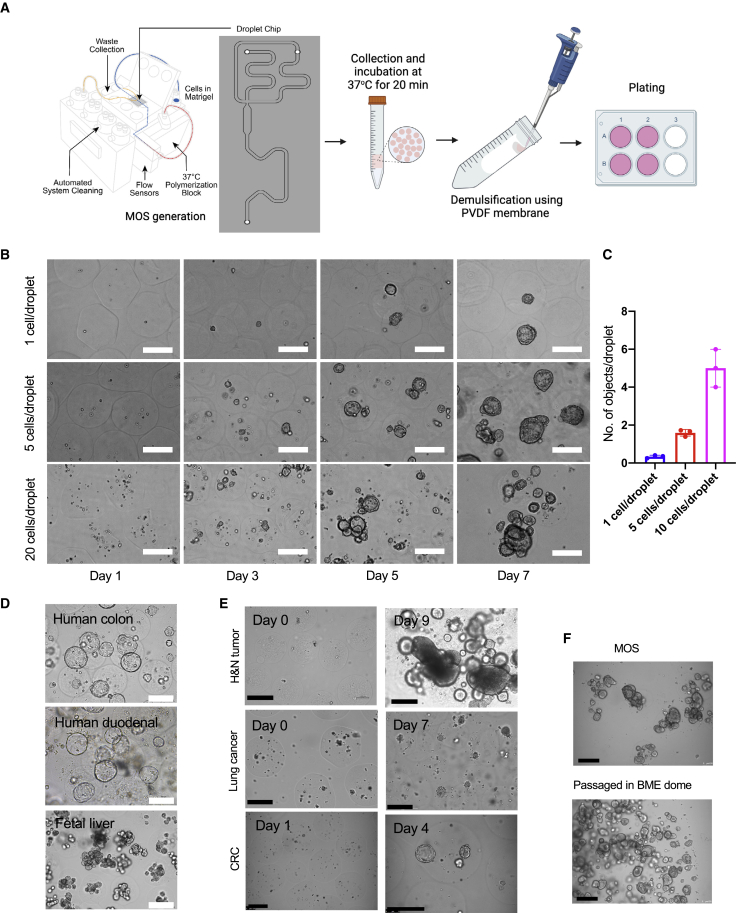
Establishment of MOS (A) The schematic of MOS generator, chip design, and the workflow of the PVDF-based demulsification. (B) Representative images of CRC MOS at different cell seeding densities over the course of a week (scale bar: 200 μm). (C) The bar graphs showing the comparisons of the average number of tumorspheres per droplet established from three different initial cell densities. (D) Representative images of established MOSs derived from human colon, human duodenal, and fetal liver lines (scale bar: 200 μm). (E) Representative images of MOSs derived from human H&N tumor tissue, lung cancer, and CRC liver metastasis core needle biopsy (scale bar: 200 μm). (F) Representative images of MOSs before and after passaging into BME dome (scale bar: 200 μm). Scale bars: 200 μm.

We designed a microfluidic device to generate hundreds to thousands of nanoliter-sized MOS droplets per minute by mixing the desired number of cells with the BME (e.g., Matrigel or Cultrex), and then loading this mixture onto the microfluidic device. Figure 1A shows the basic designs of the device core unit and the microfluidic chip. Key device features include a cooling system to prevent BME solidification during MOS generation and a heating module to accelerate the BME solidification rate in the MOS recovery vessel. A high-speed camera was incorporated to monitor MOS production in real time. The oil and sample flow rates inside the channels were generated and controlled by two separate self-contained pumps. Two oil channels meet the sample channel on the flow focusing junction and, therefore, pinch the cell mixtures to form droplets in the microfluidic chip. The droplet size is adjustable (200–500 μm in diameter) by changing the oil and sample flow rates accordingly. There is no dead volume involved in droplet generation; the minimal volume of the BME cell mixture we tested on this device was 10 μL. Additionally, we also developed a novel, chemical-free demulsification method using a hydrophobic polyvinylidene difluoride (PVDF) membrane to rapidly and efficiently separate the hydrogel-in-oil emulsion (Figure 1A, right panel) of the solidified MOS to allow nutrient and oxygen exchange. Representative images of the MOS before and after demulsification (Figure S1A) confirmed the success of this procedure. We then compared the PVDF method with two previously reported demulsification methods, the anti-static gun (Karbaschi et al., 2017) and 1H,1H,2H,2H-perfluoro-1-octanol (PFO) chemical demulsification methods (Mazutis et al., 2013). The PFO and PVDF methods efficiently removed oil from the droplets, as demonstrated by individually separated droplets in the well (Figure S1B). However, there were many visible chemical droplets (indicated by red asterisks) in the media after PFO demulsification. The anti-static gun removed minimal amounts of oil from the MOS droplets, exemplified by a big clump of droplets (circled red) also presented in the undemulsified group. To evaluate the biological compatibility of our demulsification method, we derived MOSs from human induced pluripotent stem cells (iPSCs) and monitored their growth following PFO or PVDF demulsification methods, respectively. On day 3, 20% of iPSCs MOSs were found dead in the PFO group (Figure S1C, red asterisks) versus only 5% in the PVDF group (Figure S1D), suggesting that the PVDF-based demulsification is more biocompatible with MOS culture.

Next, we evaluated the speed of establishment of MOS cultures from a colorectal cancer (CRC) PDX line. To generate MOSs, cells were encapsulated in droplets at three different initial seeding densities (1, 5, and 20 cells/droplet). Rapid establishment was observed at all three densities on day 3 after MOS generation, and typical lumen structures were observed on days 5 and 7 (Figure 1B). There was a positive correlation between cell seeding density and established tumorsphere per MOS (Figure 1C). Additionally, we successfully generated MOSs from normal tissue-derived organoid models, such as human colon, duodenum, and fetal livers. These models preserved similar morphologies (Figure 1D) and key histopathological characteristics compared with the corresponding bulk organoids. Specifically, the bulk organoids and MOSs from the fetal liver showed similar expressions of albumin and hepatocyte nuclear factor 4 alpha (HNF4A) (Figure S2A). Furthermore, when cultured as MOSs, human small intestine and duodenum organoids retained their differentiation capacity toward multiple cell types, including goblet and neuroendocrine cells (Figures S2B and S2C). We also observed the rapid establishment of MOS from head and neck squamous cell carcinoma (HNSCC), lung cancer resections, and an 18G core needle biopsy of CRC liver metastases (Figure 1E). Like bulk organoids, MOSs demonstrated the ability to be cryopreserved and passaged (Figure 1F).

Conventional bulk organoid culture requires significant manual labor and is challenging to scale up and automate for high-throughput drug screening. From a single 500 μL run of BME cell mixture, we could generate ∼35,000 MOSs (∼300 μm in diameter). Coupled with an automated MOS-dispensing system (Video S1), we were able to dispense MOSs into 1,750 wells at a density of 20 MOSs per well in less than 30 min.

Video S1. An automated MOS dispensing platform

### MOS characterization

**Figure 2:**
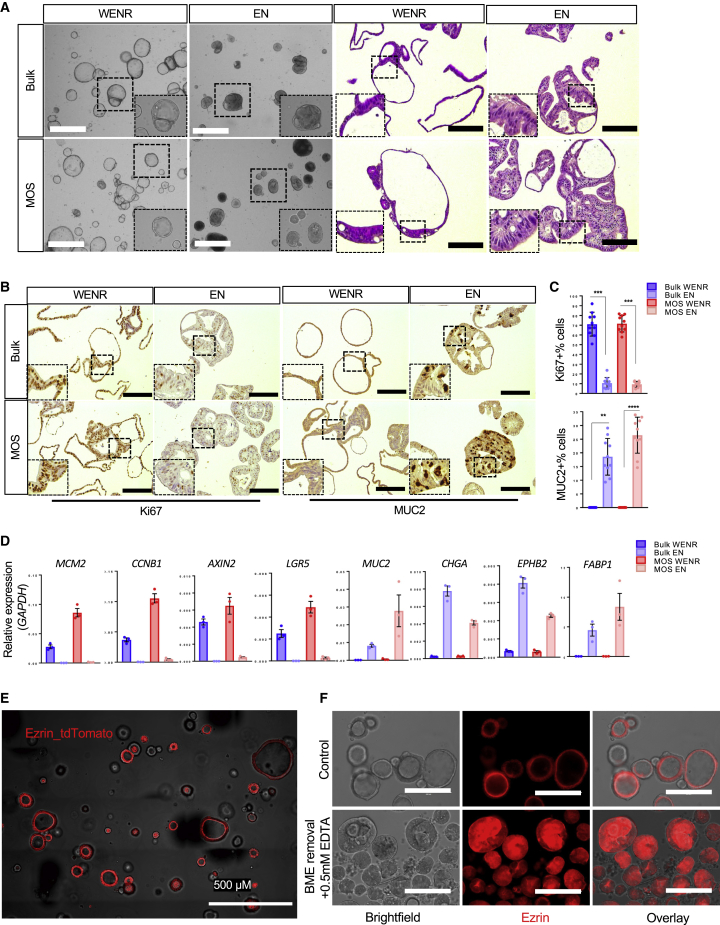
Characterization of MOSs (A) Representative bright-field images of human colon bulk organoids and MOSs in expansion (WENR) and differentiation (EN) medium accompanied by H&E images of the same cultures (scale bar: 100 μm). (B) Representative staining of Ki67 and MUC2 for human colon bulk organoids and MOSs cultured in WENR and EN medium. (C) Bar graphs showing the quantification of the MUC2 and Ki67 staining (bars show the mean ± SEM of n = 10 different images). (D) Bar graphs representing gene expression of selected markers for MOS and bulk cultures in WENR versus EN medium (bars show the mean ± SEM of n = 3). (E) Representative confocal image of Ezrin reporter MOS (scale bar: 500 μm). (F) Representative images of the Ezrin reporter MOS under normal culture condition (top) or after BME removal (bottom) (scale bar: 200 μm).

To determine if MOSs maintain the critical features of bulk organoid cultures, we first used an organoid line derived from human intestine tissue to assess the morphology and expression levels of several key markers of MOSs and bulk organoids in expansion and differentiation media conditions. MOSs and bulk organoids exhibited similar morphological changes (observed by both bright field and H&E) in response to the differentiation medium (Figure 2A). Depletion of Wnt ligands and R-spondin1 (Rspo1) reduced proliferation, as indicated by Ki67 staining, and reduced expressions of proliferative markers *MCM2* and *CCNB1* while inducing differentiation of both MOSs and bulk organoids into mucus-producing goblet cells, as indicated by MUC2 staining (Figures 2B and 2C). Accordingly, expression of the Wnt target genes *AXIN2* and *LGR5* decreased upon differentiation, but levels of differentiation markers such as *MUC2*, *CHGA*, *EPHB2*, and *FABP1* increased (Figure 2D). The protein expressions of CHGA, VILLIN, and EPHB2 were confirmed by immunohistochemical (IHC) staining (Figure S3). We similarly compared endometrial MOSs with bulk organoids. Hormonal treatment induced comparable changes in MOS and bulk organoid cultures (Figures S4A and S4B). Estrogen priming for 2 days followed by 7 days of progesterone treatment induced differentiation toward ciliated cells, marked by the expression of acetylated α-tubulin (Boretto et al., 2019) (Figure S4C; Video S2, ciliated cells indicated by circles). To examine organoid polarity in MOSs, we used CRISPR-Cas9-mediated homology-independent organoid transgenesis (CRISPR-HOT) (Artegiani et al., 2020) to tag a human colon organoid model with a fluorescence reporter tethered to apically expressed Ezrin. tdTomato-tagged Ezrin was enriched in the apical side of intestinal MOSs (Figure 2E), suggesting that MOSs preserved the basal-out polarity. Upon BME removal and EDTA treatment, the polarity was reversed to apical out (Figure 2F). These data demonstrated that MOSs maintain the key characteristics of organoids grown in bulk culture and are potentially viable *ex vivo* models to study cellular physiology and diseases.

Video S2. Cilia movement in differentiated endometrial bulk organoids (left) and MOS (right)

### MOSs enable uniform nutrient accessibility for studying nutrient dependence

**Figure 3:**
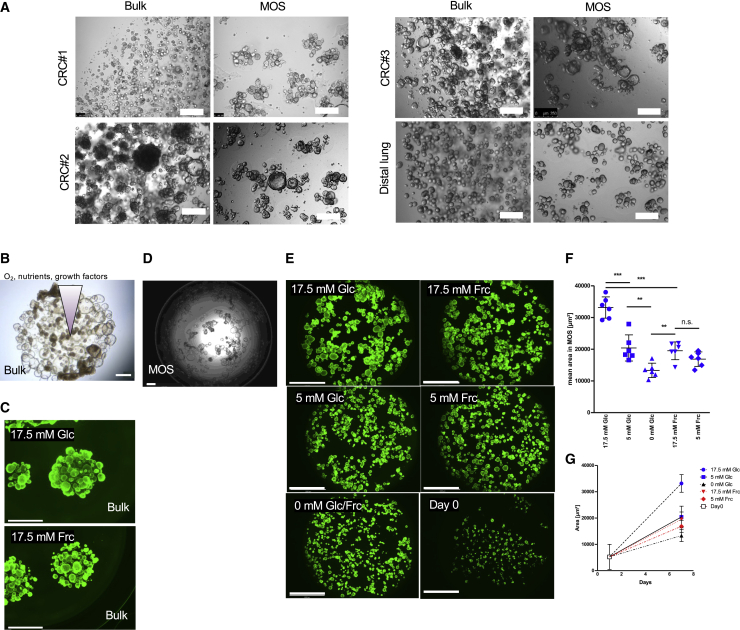
MOSs enable uniform nutrient accessibility (A) Representative images of CRC#1, CRC#2, CRC#3, and a distal lung line growing in bulk organoid cultures or as MOSs (scale bar: 200 μm). (B) Duodenum organoids in BME dome in presence of 17.5 mM glucose (scale bar: 500 μm). (C) Duodenum organoids cultured in BME domes were stained with CAM (scale bar: 2,000 μm). (D) Duodenum organoids cultured in MOS in presence of 17.5 mM glucose (scale bar: 500 μm). (E) Representative images of duodenum MOSs after 0 or 7 days of treatment with different glucose (Glc) or fructose (Frc) concentrations (scale bar: 2,000 μm). MOSs were stained with CAM. (F and G) Image-based quantification of the green area visualized for each condition as mean values ± SD (n = 6 from two biological replicates) after 7 days (F) or over time (G).

A recent study reported that the spatiotemporal gradient of Wnt3a inside the BME dome causes inter-organoid heterogeneity of mouse intestine organoids in conventional organoid culture (Shin et al., 2020). The small size and large surface-to-volume ratio of MOSs may enable more efficient and uniform diffusion of growth factors, nutrients, and oxygen versus the BME dome. We observed that MOSs had a growth advantage, as demonstrated by the larger sizes of organoids and less necrotic organoids, than their counterparts in BME domes from the three CRC PDO models and one distal lung-derived model (Figure 3A). This observation suggests that studying nutrient dependence using bulk organoid cultures in BME domes may pose a challenge because the organoid nutrient accessibility gradient could overlap with nutrient utilization phenotypes and mask their effects. Correspondingly, when treating duodenum organoids in BME domes with two different carbohydrate diets, namely fructose and glucose, capturing quantifiable changes in the individual organoid sizes in the dome was challenging due to the heterogeneous organoid morphologies (smaller/necrotic organoids in the center versus larger organoids on the edge) within BME dome (Figures 3B and 3C). In contrast, MOS culture diminishes nutrient gradients and overlapping structures (Figure 3D and 3E), which leads to accurate image-based quantification of the calcein-AM (CAM)-stained average duodenum organoid area over time (Figure 3F–3G). Utilizing MOSs in this manner revealed that 17.5 mM fructose significantly decreased organoid size compared with the same glucose concentration. Furthermore, as expected, reducing the glucose concentration also reduced the organoid volume significantly. However, the organoid area was not impacted by the reduction of fructose concentration from 17.5 to 5 mM and only decreased when depleted completely. These results suggest that MOS culture provides a more quantitative approach for assessing nutrient dependence.

### MOSs enable efficient viral infections and CAR-T cell infiltration

**Figure 4:**
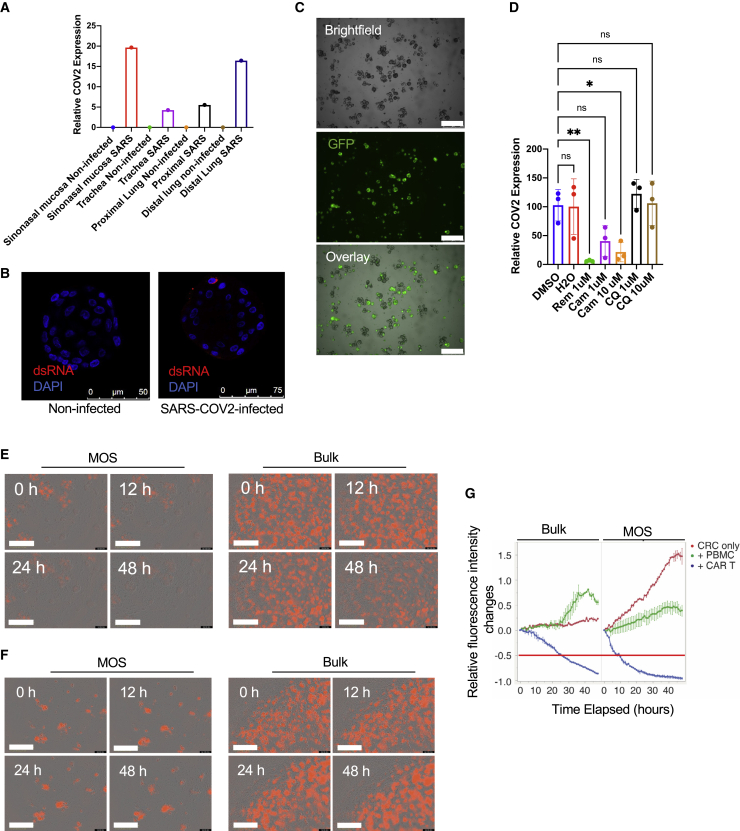
MOS-based assays for viral infections and assessing CAR-T cell-mediated cytotoxicity (A) qRT-PCR measures the SARS-CoV-2 expression in the airway MOSs after 48 h of infection. (B) Representative images of double-strand RNA (dsRNA) immunofluorescence (IF) staining of non-infected and SARS-CoV-2-infected airway MOSs. (C) Representative images of airway MOSs after influenza infection for 24 h. GFP-positive spots indicate the influenza-infected MOSs (scale bar: 500 μm). (D) qRT-PCR measures the SARS-CoV-2 expression in sinonasal MOSs in response to the treatments of remdesivir, camostat, or CQ. ^∗^p < 0.05; ^∗∗^p < 0.01; ns, not significant (bars show the mean ± SEM of n = 3 biological replicates). One-way ANOVA was used to determine the statistical significance. (E) Representative images from co-culture of HER2^+^ CRC MOSs (left) or bulk organoids (right) with anti-HER2 CAR-T cells over a 48 h period (scale bar: 500 μm). (F) Representative images from co-culture of HER2^+^ CRC MOSs (left) or bulk organoids (right) with PBMCs over a 48 h period (scale bar: 500 μm). (G) Time-course data from IncuCyte S3 for red fluorescent signal with bulk organoid comparison. The red horizontal line indicates the 50% decrease of red fluorescence intensities compared with time 0.

Conventional organoids in BME domes require dissociation into single-cell suspensions or small fractions for efficient viral infection. Here, we tested whether the MOSs’ small size and large surface-to-volume ratio facilitate enhanced viral infection rates of cells compared with bulk organoid cultures. We grew a CRC PDO as MOSs and bulk organoids and directly infected them using adeno-associated virus (AAV; AAV8-CBh-scGFP) and influenza (Flu A/California/2009_GFP). We observed a significant increase of GFP-positive cells in the MOSs but not in the BME dome 36 h post-infection (Figure S5A). Next, we then directly infected MOS derived from autopsied human respiratory tract, including the sinonasal mucosa, trachea, proximal lung, and distal lung, with SARS-CoV-2 or influenza. After 48 h of SARS-CoV-2 infections, we observed a significant increase in the SARS-CoV-2 qPCR signals in MOS infected with SARS-CoV-2 but not in uninfected MOS controls (Figure 4A). Successful SARS-CoV-2 infection was confirmed by immunofluorescence staining with double-stranded RNA (dsRNA) (Figure 4B). Influenza-infected MOS exhibited a strong increase in GFP-positive cells 24 h post-infection (Figure 4C) and substantial cell death 48 h post-infection (Figure S5C). We then tested three anti-SARS-CoV-2 compounds on our infected sinonasal mucosa MOSs: remdesivir (an RdRp inhibitor), camostat (a TMPRSS2 inhibitor), and chloroquine (CQ). All three drugs did not show obvious effects on cell viability (Figure S5B). Remdesivir had the strongest inhibitory effect on SARS-CoV-2 replication at 1 μM, as measured by the SARS-CoV-2 qPCR assay. Camostat greatly inhibited SARS-CoV-2 replication at 10 μM but had only a modest effect at 1 μM. CQ did not show any significant effect (Figure 4D), which was consistent with a report that CQ showed an effect only in Vero cells but not in human lung cells (Hoffmann et al., 2020). Overall, these results suggest that MOSs may serve as an efficient model for studying host-pathogen interactions and for anti-viral drug screening.

Tumor immune co-cultures using PDOs have been demonstrated for immuno-oncology studies and as a potential *ex vivo* model for cell therapy (Cattaneo et al., 2020; Dijkstra et al., 2018; Michie et al., 2019; Neal et al., 2018). However, akin to challenges for direct viral transduction, the ability of patient-derived immune cells to uniformly penetrate BME domes and interact with bulk organoids for robust, quantitative readouts has been a challenge. We tested whether the small size of MOS, which is approximately the diffusion limit of vascularized tissue, allows more efficient penetration of CAR-T cells and leads to a rapid killing assay. A CRC organoid line was transduced with lentivirus encoding mCherry-HER2 and sorted by flow cytometry. The transduced line was then grown in bulk organoid culture or MOSs and co-cultured with either CAR-T cells specific to HER2 or non-transduced PBMCs. The T cell-killing ability was monitored in real time using Incucyte S3 (see Video S3). Greater immune cell penetration was observed (Figure 4E) in MOSs versus in bulk organoids following 12 h of co-culture. Consistently, a dramatic decrease (∼60%) in cell viability (red fluorescence signal) was observed in the HER2^+^ CRC MOSs co-cultured with anti-HER2 CAR-T cells versus a slight decrease (∼20%) of the red HER2^+^ fluorescence signal observed in the bulk organoid culture (Figure 4G). A further decrease in red fluorescence signal was observed in MOSs after 24 and 48 h of co-culture of anti-HER2 CAR-T cells (Figure 4E), whereas there was an increase in red fluorescence signal over time in HER2^+^ CRC MOSs co-cultured with PBMCs (Figure 4F). This proof-of-concept study suggests that MOSs’ unique attributes may be adapted for developing novel three-dimensional (3D) model-based immuno-oncology assays.

Video S3. Incucyte movie shows the growth of HER2+ CRC MOS alone (top), co-culture with PBMC (medium) or HER2 CAR-T

### MOSs recapitulate similar treatment responses to chemo-, targeted, and radiation therapies compared with bulk organoids

**Figure 5:**
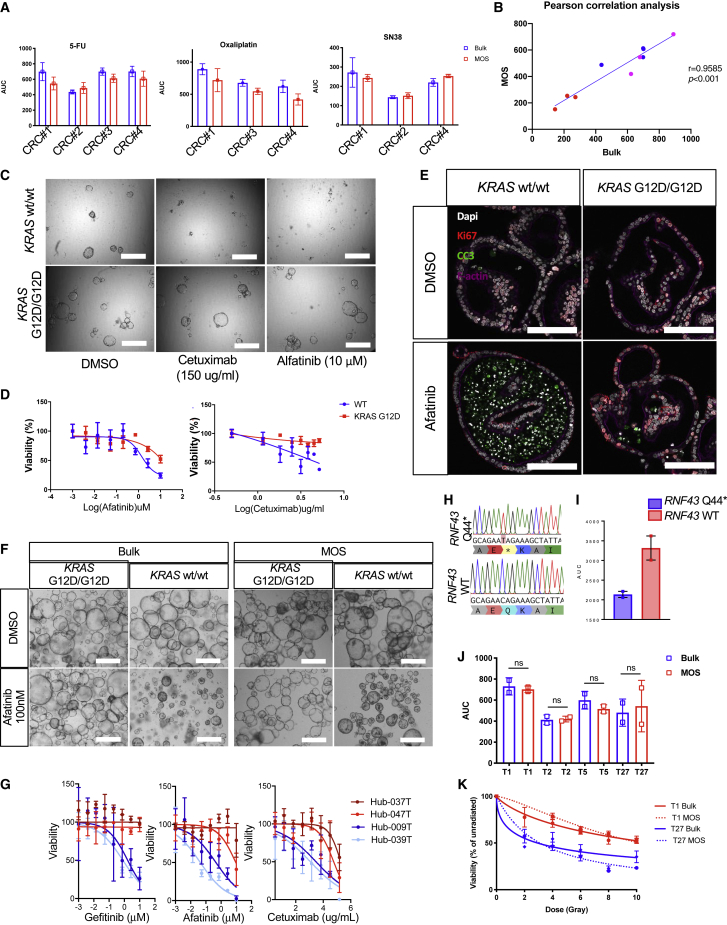
Response to chemo-, irradiation, and targeted therapy (A) AUCs of the CRC organoid cultures generated as bulk organoids or MOSs after being exposed to increasing dosages of 5-FU, oxaliplatin, and SN38 in CRC lines (error bars show the mean ± SEM, n = 3). (B) The linear correlations of the AUC detected from bulk and MOS treated conditions. Red dots, SN38; blue dots, 5-FU; magenta dots, oxaliplatin. (C) Representative images of the paired *KRAS* isogeneic lines treated with vehicle or cetuximab and afatinib (scale bar: 100 μm). (D) The dose-dependent drug response curves of the paired *KRAS* isogenic lines in response to the treatments of cetuximab and afatinib; (error bars show the mean ± SEM, n = 3). (E) Ki67 and cleaved caspase 3 (CC3) co-staining showing decreased proliferation and increased apoptosis for *KRAS* wild-type (WT) MOSs treated with 100 nM afatinib for 3 days (scale bar: 100 μm). (F) Bright-field images of bulk organoids and MOSs treated with 100 nM afatinib for 3 days (scale bar: 100 μm). (G) The dose-dependent drug response curves of four CRC PDO cultures generated as MOSs after exposure to increasing dosages of cetuximab, afatinib, and gefitinib (bars show the mean ± SEM, n = 3). (H) Targeted sequencing of a portion of the exon 1 of *RNF43* showing one sample with a C>T transition resulting in a Q44^∗^ mutation. (I) Bar graphs showing the AUCs calculated for the IWP2 drug screen performed on *RNF43* WT and *RNF43* mutant lines, the latter of which is highly sensitive to the compound (bars show the mean ± SEM of two independent biological replicates). (J) AUC of four PDOs generated as MOSs (red) or bulk organoids (blue) after exposure to increasing dosages of irradiation. (K) Dose-response curves displaying viability of two PDOs generated as bulk organoid (solid line) or MOSs (dotted line) exposed to increasing dosages of irradiation. PDOs were originally generated from patients with HNSCC that relapsed (red) or did not relapse (blue) clinically to treatment with irradiation. Bars show the mean ± SD. n = 3, and each experiment was repeated twice. Paired t test was used to determine no significance (ns).

PDOs have been shown to successfully correlate *in vitro* drug readouts with clinical response to treatment (Driehuis et al., 2019a; Kim et al., 2021; Narasimhan et al., 2020; Ooft et al., 2019; Yao et al., 2020). Here, we examined whether MOSs maintain similar predictability as bulk organoids to various classes of drug treatments. Bulk organoids and MOSs responded similarly to three standard-of-care chemotherapy reagents, 5-FU, SN38, and oxaliplatin, in all four CRC PDO models tested as determined using the CellTiter-Glo 3D (CTG) viability assay, and the areas under the dose-response curve (AUCs) were not significantly different (Figures 5A and 5B). A human colon organoid model (parental) and its isogenic (*KRAS* G12D) model (Drost et al., 2015) were included to test the responses of MOSs to two anti-EGFR targeted reagents (Chong and Janne, 2013): (1) cetuximab, a chimeric monoclonal antibody that binds to and inhibits EGFR, and (2) afatinib, which inhibits both EGFR and Her2. The MOSs derived from the parental model were more sensitive to both drugs than the MOSs derived from the isogenic line with the *KRAS* G12D mutation (Figures 5C–5E). In four CRC patient-derived models, including two *KRAS* wild- type and two *KRAS* mutant models (van de Wetering et al., 2015), *KRAS* wild-type MOSs were more sensitive to anti-EGFR treatments than the two *KRAS* mutant MOSs (Figure 5G). *RNF43* mutations, commonly detected in CRC (Giannakis et al., 2014), enhance the sensitivity of tumor cells toward the porcupine inhibitor IWP2 (van de Wetering et al., 2015). Here, we also observed that *RNF43* mutant MOSs exhibited higher sensitivity to IWP2 treatment compared with *RNF43* wild type (Figures 5H and 5I). Additionally, MOSs established from four HNSCC PDO models were exposed to increasing doses of irradiation. AUCs for MOS and PDO models (as determined using CTG) were not significantly different (Figure 5J). Two of the patients had subsequent radiation therapy in the clinic. One patient relapsed (red), and the other did not (blue), which was consistent with MOS and PDO readouts (Figure 5K). Overall, these data suggest that MOSs provide a robust alternative for generating clinically relevant, patient-focused data in an *ex vivo* setting.

### MOS technology is compatible with high-throughput imaging and provides a more robust drug assay

**Figure 6:**
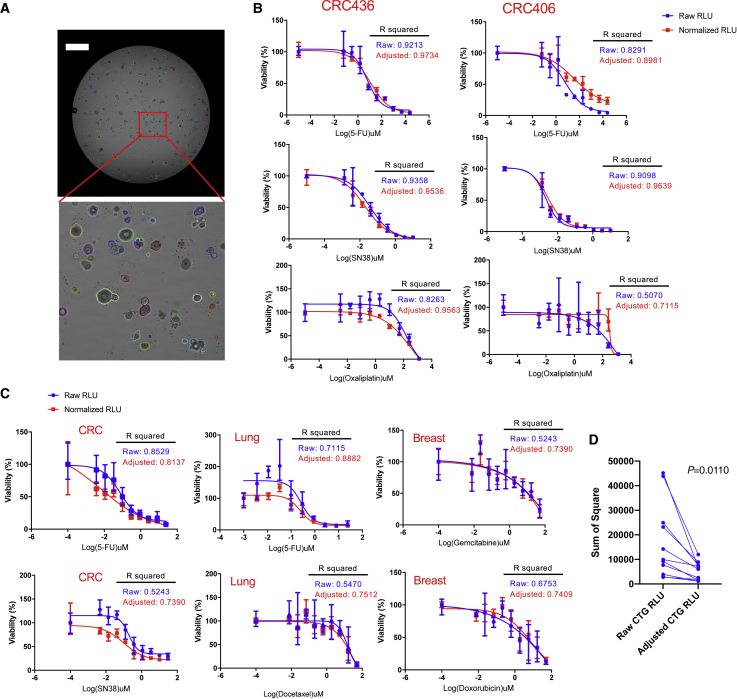
MOS coupled with machine learning enables a tSA-based normalization strategy for improving the robustness of bulk drug assay (A) A representative image of a whole-well scanning and a zoom-in view showing the segmented objects detected by in-house machine-learning algorithm (scale bar: 1,000 μm). (B) Comparisons of drug response curves measured by CTG assay before and after normalization with day 0 tSA in two established CRC PDO lines. CRC406 and CRC436 MOS were treated with SN38, 5-FU, and oxaliplatin for 3 days. The blue curve shows the unnormalized data points, and the red curve shows the data points after normalization. The error bars indicate the ranges of the data derived from three independent replicates. (C) Comparisons of drug response curves measured by CTG assay before and after normalization with day 0 tSA in lung, CRC, and breast cancer primary tissue-derived MOSs. The lung MOS line was treated with 5-FU or docetaxel for 3 days. The CRC MOS line was treated with 5-FU or SN38 for 3 days, and the breast MOS line was treated with gemcitabine or doxorubicin for 3 days. The blue curve shows the unnormalized data points, and the red curve shows the data points after normalization. The error bars indicate the ranges of the data derived from three replicate wells. (D) Comparison of the difference between the means; each point of data is indicated by the sum of square after normalization (p = 0.011, paired two-tailed t test).

The often-limited number and highly variable size/growth pattern of PDO/tumorspheres introduce significant well-to-well variations, and thus, pose readout challenges for conventional drug assays using 3D cell culture models. Unlike the multifocal planes that are required for thorough analysis when growing bulk organoids in a BME dome, we noticed that most MOSs were settled on the bottom of the micro-plate wells (e.g., 96- or 384-well plates) and did not overlap with other MOSs after dispensing (Figures S6A and S6B). This feature allowed us to image individual MOSs at the whole-well level without requiring multiple z stack scans using a micro-well-plate-based image cytometer (Celigo Imaging Cytometer). Coupled with rapid imaging, we re-trained the implementation of Mask-RCNN (He et al., 2017) image segmentation framework available on Detectron2 (Kirillov et al., 2020) and developed an in-house algorithm to accurately segment and measure the surface areas of organoids/tumorspheres from the acquired bright-field images (Figure 6A). A positive correlation of the total surface area (tSA) of MOSs with CTG luminescence signals was observed in two CRC models at the well level (Figures S6C and S6D), suggesting that we can use the tSA extracted from bright-field images to normalize the endpoint CTG values and, therefore, reduce the variations caused by plating or heterogenous growth patterns of MOSs when performing a drug assay. To validate this normalization strategy, we further treated two CRC MOSs each with SN38, 5-FU, or oxaliplatin. After normalizing the raw RLU values of CTG assay with the day 0 tSA, the range of error bars of three replicate wells were reduced; also, R squared of the adjusted CTG values were significantly improved (Figure 6B), thus confirming that this image-based day 0 tSA normalization strategy indeed improved the robustness of CTG-based drug assays. We further tested this normalization strategy in the p0 primary tumor-derived MOSs (derived from a lung cancer, a CRC, and a breast cancer) treated with drugs within 7 days of establishment. We observed variable size of tumorsphere in p0 MOSs in all three lines (Figure S6A) and significant CTG readout variation within triplicate wells (Figure 5C, blue curves). Using our tSA normalization strategy, we observed a significant reduction of range error bars and an increase of R squared (Figure 6C, red curves) of the adjusted CTG values across all conditions. The sum of the square, which measures the deviation of data points from the mean value, were also significantly decreased after normalization with tSA (Figure 6D). These data suggest that the MOS platform enables an image-based normalization strategy to increase the robustness of the CTG-based and other whole-well bulk drug assays.

### MOSs enable an AI-based orthogonal drug assay to differentiate cytostatic versus cytotoxic drug effects and capture heterogeneous treatment response at single-organoid/tumorsphere resolution

**Figure 7:**
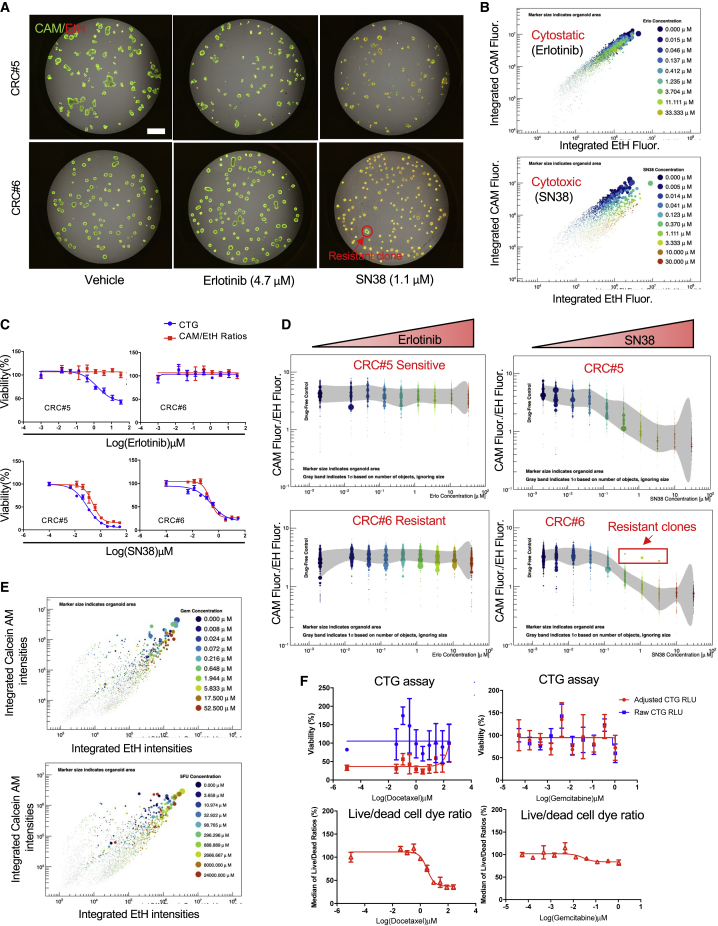
An orthogonal AI-based analysis approach to differentiate the cytostatic/cytotoxic drug effects and capture heterogeneous drug response at individual-tumorsphere/organoid resolution (A) Representative images of two CRC MOS treated with vehicle, erlotinib, or SN38, co-stained with live cell dye (CAM) and dead cell dye (EtH). The red circle highlights a resistant clone discovered in CRC#6 treated with SN38 (scale bar: 1,000 μm). (B) Scatterplots show the differential drug responsive patterns of CRC#5 treated with of erlotinib (cytostatic) or SN38 (cytotoxic) respectively at the individual organoid resolution. The size of each dot reflects the relative surface area of the individual segmented object. (C) The drug response curves of two CRC MOS models treated with erlotinib or SN38. Blue curves were plotted based on CTG assay and red curves were plotted based on the median ratios of CAM/EtH dye integrated intensities. (D) Scatterplots show the dose-dependent changes of the ratios of CAM/EtH dye integrated intensities of CRC#6 and CRC#5 treated with erlotinib or SN38, respectively. The red rectangle highlights the drug-resistant clones identified in the CRC#6 treated with SN38. The x axis indicates the range of drug concentrations. The size of each dot reflects the relative surface area of the individual segmented object. Gray band indicates 1σ based on number of objects, ignoring size. (E) Scatterplots show how the individual tumorspheres responded to 3 days of gemcitabine or 5-FU treatment in a primary lung tumor-derived MOS. (F) The comparisons of drug response curves measured by CTG assay (top panel) versus median of live/dead cell dye ratios (bottom panel) in a sarcoma primary tissue-derived MOS.

Bulk endpoint assays such as CTG alone are unable to capture heterogeneous treatment response, distinguish cytotoxic versus cytostatic effects, or delineate tumorspheres from stromal cells, all of which are important for assessing clinical drug response. To further enhance the resolution and power of the MOS-based drug assay, we developed an orthogonal approach by incorporating a combination of live (CAM) and dead (Ethidium Homodimer II, EtH) cell dye staining to the CTG drug assay. We tested this approach on two CRC MOS models treated with SN38 or erlotinib (an EGFR inhibitor). Using our in-house artificial intelligence (AI) algorithm, we were able to automatically capture different readouts (size, integrated live/dead cell dye signals) from the acquired images (Figure 7A) and track the drug response at individual organoid/tumorsphere resolution. We observed that SN38 treatment caused a dose-dependent increase of integrated EtH and a decrease of integrated CAM intensities in both models (Figure 7B; Videos S4, and S5), but no change was observed in response to erlotinib treatment. By integrating the size and the ratios of integrated fluorescence intensities of CAM and EtH (CAM/EtH ratios) of each segmented object, we could track the drug responses on a more granular level and potentially differentiate the cytotoxic versus cytostatic drug effects. We observed that SN38 caused decreases of both size and CAM/EtH ratios in both models, whereas erlotinib only caused size decrease in CRC#5 but no obvious change of CAM/EtH ratios (Figure 7D), suggesting that SN38 is a cytotoxic drug but erlotinib is a cytostatic drug. Upon further plotting the drug response curves using the median values of CAM/EtH ratios from each well (Figure 7C), we observed comparable drug response curves in both models when treated with SN38 compared with the CTG-based assay. However, there was a divergence between CTG plot and CAM/EtH ratio plot in CRC#5 treated with erlotinib, thus further confirming that erlotinib triggered cytostatic effect on CRC#5. Interestingly, we also identified several outliers on CAM/EtH ratio plot (Figure 7D, highlighted in the red rectangle) in CRC#6 model treated with SN38; these outliers turned out to be drug-resistant clones, as evidenced by the fluorescence images (Figure 7A). Therefore, the CAM/EtH cell dye ratios coupled with size measurement and CTG allowed us to differentiate the cytotoxic versus cytostatic drug effects and capture the heterogenous drug responses. Given the heterogenous cell composition and growth patterns on the p0 MOSs, a more heterogenous distribution patterns of CAM/EtH ratio plots were observed in the aforementioned primary lung tumor-derived p0 MOSs (Figure 7E; Video S6) when treating with 5-FU and gemcitabine. Moreover, in MOSs derived from sarcoma tissue and treated with docetaxel or gemcitabine on day 7 after establishment, the bulk CTG readouts, raw CTG or adjusted CTG, were not able to provide any meaningful dose-dependent drug response curves due to the limited number of tumorspheres, notable resident stromal cells in the MOS droplet (Figure S6F), and well-to-well variation (Figure 7F, top panel). Conversely, the median values from the live/dead cell ratios detected and measured from the same well showed a clear dose-dependent drug response to docetaxel (Figure 7F, bottom panel) and reduced sensitivity to gemcitabine treatment. These data suggest that our orthogonal image-analysis-based MOS drug assay better delineates MOS 3D structures from individual stromal cells. Importantly, each individual tumorsphere within MOSs is captured, which provides a unique set of data points and can, therefore, overcome the fundamental limits (e.g., low cell number, well-to-well variation, heterogeneity, and signal-to-noise ratio) of bulk assays to enable clinical precision oncology within a short time frame.

Video S4. The animated scatter plot shows the response of CRC#5 to SN38 or erlotinib treatment at single tumorsphere resolution

Video S5. The animated scatter plot shows the response of CRC#6 to SN38 or erlotinib treatment at single tumorsphere resolution

Video S6. The animated scatter plot shows the response of lung cancer derived primary MOS to gemcitabine or 5-FU treatment

## Discussion

Although functional precision medicine has attracted substantial interest, the speed, throughput, complexity, and reproducibility of current patient-derived models have limited potential clinical applications. Here, we coupled a novel microfluidics device and chemical-free demulsification approach to uniquely generate thousands of miniaturized MOSs for different tissue types. Our microfluidic system has minimal dead volume and can run up to four samples simultaneously. By integrating an automated MOS-dispensing system and a micro-well plate imager, we established a scalable and high-throughput MOS-based drug-screening pipeline. MOSs provide several advantages compared with conventional 3D bulk organoid culture, including scalability, high throughput, automation, and ease of imaging. MOSs exhibit similar characteristics (morphology, polarity, and differentiation potential) and therapeutic responses (chemotherapy, targeted, and irradiation) versus bulk organoids.

MOSs can be rapidly established and assayed with reduced material, reagents, and labor. This is in direct contrast to organoid generation, which involves continuously expanding organoids up to passage three or five to have enough material to perform a drug screen. MOSs, which retain original tissue composition and cellular properties, do not require multiple passaging and can be screened immediately upon establishment. Our recent published study demonstrated that a rapid MOS drug assay based on an 18G biopsy reliably predicted the clinical responses of patients with CRC liver metastasis to chemotherapy within 14 days (Ding et al., 2022). Furthermore, extrapolating to other types of assays for a variety of indications, MOSs allow uniform nutrient accessibility, direct viral infection, and efficient T cell infiltration due to small size and large surface-to-volume ratio. For proof of principle, we tested the dietary effect of different sugars, directly infected airway MOSs with SARS-CoV-2 and influenza to assess potential anti-SARS-CoV2 drugs, and developed a rapid functional assay for assessing the potency of CAR-T cells against tumor cell-derived MOSs. Lastly, we developed a machine-learning-enhanced, high-throughput imaging assay that can capture drug-resistant clones and overcome the limitations of small sample size, well-to-well variation, and tissue heterogeneity. By multiplexing the CTG assay with a live/dead cell dye staining followed by a deep-learning-based, high-throughput image analysis, we were able to capture a variety of readouts (bright field, fluorescence, and CTG luminescence) from the same set of samples and measure the drug response at both whole-well level and the individual-tumorsphere level. The tSA normalization strategy allows us to mitigate the organoid plating variations for bulk assay measurements. In addition, we demonstrated that CAM/EtH staining combination with CTG assay allows us to further differentiate the cytostatic versus cytotoxic effects and, in addition, capture the heterogeneous drug response at a single-tumorsphere resolution.

There are two major challenges when performing drug assays on primary tumor-derived models at p0, which could better guide clinical decision-making: (1) lack of adequate tumorspheres established from p0, and (2) difficulty differentiating tumor-specific treatment response readouts confounded by the presence of stromal cells. By developing an MOS-based machine-learning algorithm, we were able to capture treatment responses at the individual-tumorsphere level and differentiate tumorshperes from stromal cells. Moreover, the ability to treat each tumorsphere within an MOS as a biological replicate dramatically increased the statistical power of the assay. This attribute is critical feature of MOS technology given that the clinical biopsies are usually small with limited cell numbers. Henceforth, MOSs can be used as both diagnostic assays to guide patient treatment and as screening platforms for new anti-cancer and anti-viral drug discovery efforts.

## Experimental procedures

### Chip design and MOS droplet generation

Our MOS generation chips were fabricated in a co-polymer plastic to provide robust and desirable surface properties. The BME droplets were generated using the MOS generator as described in the results. Oil (QX200 Droplet Generation Oil for EvaGreen#1864005) and sample flow inside the channels were controlled by two separate pumps. The initial number of cells per droplet could be controlled by adjusting the cell densities in the BME. The generated droplets were then collected and incubated at 37°C to solidify the BME droplets.

### MOS droplet demulsification

The chemical and anti-static gun demulsification methods were conducted as previously described (Karbaschi et al., 2017). For the membrane demulsification method, the solidified BME droplets were transferred onto a hydrophobic PVDF membrane. After 2–3 min of incubation (to allow the oil to evaporate and be absorbed by the membrane), the demulsified MOSs were washed from PVDF using the culture media and moved to culture vessels.

### Bulk organoid and MOS cultures

The BME we used in this study were either Corning Matrigel matrix or Cultrex. Briefly, human small intestinal cells were processed and cultured as described previously (Beumer et al., 2018; Ishikawa et al., 2011). Human colon organoids were cultured in WENR medium. Differentiation of human colon and small intestinal organoids was induced by removing Wnt surrogate and Rspo1 conditioned medium (EN) for 5–7 days. Endometrial organoids were established from human endometrium as previously described (Boretto et al., 2019) and were differentiated by sequentially treating the organoids with 10 nM 17-β, estradiol, 1 μM cAMP, and 200 ng/mL of progesterone. The culture medium for airway organoids (Sachs et al., 2019) and human H&N cancer PDOs were processed and cultured as previously described (Driehuis et al., 2019b). Green fluorescent protein (GFP)-labeled feeder-free human iPSCs were purchased from Angio-Proteomie, and iPSC MOSs were cultured using iPSC serum-free media (cat. no. cAP-50). All medium information is listed in the Table S1.

### Patient samples

All tumor tissues and immediate post-mortem specimens were collected at Duke University Hospital through the Duke BioRepository & Precision Pathology Center (BRPC). The study was reviewed and approved by the Duke Institutional Review Board. Informed consent was obtained from all participants. The clinical data of patients were collected through the BRPC medical record system. IRB approvals (IRB # Pro00089222) and research protocols were approved by the relevant institutional IRBs.

### HUBRECHT ethics statement

The H&N PDO cultures used in this study were first described by Driehuis et al. (2019a). Coding in this manuscript is identical to that applied in that paper. The small intestinal organoid ileal line used in this study was derived from a human ileum and was established before (Beumer et al., 2018). The duodenal small intestinal organoid line was established from a patient with duodenal cancer. Biobank Research Ethics Committee of the University Medical Center Utrecht (TCBio) approved the biobanking protocol: 12-093 HUB-Cancer according to the University Medical Center Utrecht (UMCU) Biobanking Regulation for the head and neck and ileal study and 14-472 HUB-Ovarian for the endometrium. The CRC organoids used in this study were described in van de Wetering et al. (2015). Colonic tissues were obtained from The Diakonessen Hospital Utrecht with informed consent, and the study was approved by the ethical committee. The collection of patient data and tissue was performed according to the guidelines of the Network of Research Ethics Committees (EUREC) following European, national, and local law. All donors participating in the study signed informed consent forms and can withdraw their consent at any time, leading to the prompt disposal of their tissue and any derived material as well as the cessation of data collection. Future distribution of organoids to any third (academic or commercial) party must be authorized by the METC UMCU/TCBio at request of the HUB to ensure compliance with the Dutch Medical Research Involving Human Subjects Act.

### Data availability and additional method details

The main data supporting the results in this study and additional method details are available within the paper and its supplemental information. The raw imaging data files are available from the corresponding authors on reasonable request.

## Author contributions

Z.W., D.H., H.C., and X.S. designed the research. Z.W., M.B., R.M., N.N., E.S.R., M.N., H.Y., C.H., B.E.H., A.T.H., E.D., J.B., G.O.R., D.G., D.W., J.P., M.H.G., A.Y., A.S., E.B., S.D., and K.X. performed experiments. W.Q., R.L.v.I., and D.G. performed the data analysis. B.E.H., M.N., and A.T.H. performed the BSL3 lab work. D.D. and D.N. contributed to machine and chip design. R.A.-H., D.J., B.J.G., and C.G. collected the autopsy samples. S.B. and J.D. helped with figure preparation. N.S.H., D.H., H.C., and X.S. supervised the project. Z.W. and X.S. wrote the manuscript with input from all authors.

## Supplemental information

Document S1. Supplemental experimental procedures and Figure S1–S6

Table S1. Information of media recipes, reagents, and primers

Document S2. Article plus supplemental information
