# Supplementary material for: Rapid tissue prototyping with micro-organospheres
Source: Stem Cell Reports. 2022 Aug 18;17(9):1959–75. doi: 10.1016/j.stemcr.2022.07.016 (PMC9481922; doi:10.1016/j.stemcr.2022.07.016)
Supplement: Document S1. Supplemental experimental procedures and Figure S1–S6 [file mmc1.pdf]

Stem Cell Reports, Volume *17*

Supplemental Information

Rapid tissue prototyping with micro-organospheres

Zhaohui Wang, Matteo Boretto, Rosemary Millen, Naveen Natesh, Elena S. Reckzeh, Carolyn Hsu, Marcos Negrete, Haipei Yao, William Quayle, Brook E. Heaton, Alfred T. Harding, Shree Bose, Else Driehuis, Joep Beumer, Grecia O. Rivera, Ravian L. van Ineveld, Donald Gex, Jessica DeVilla, Daisong Wang, Jens Puschhof, Maarten H. Geurts, Athena Yeung, Cait Hamele, Amber Smith, Eric Bankaitis, Kun Xiang, Shengli Ding, Daniel Nelson, Daniel Delubac, Anne Rios, Ralph Abi-Hachem, David Jang, Bradley J. Goldstein, Carolyn Glass, Nicholas S. Heaton, David Hsu, Hans Clevers, and Xiling Shen

Supplemental experimental procedures

Immunofluorescent staining. Immunofluorescent staining was performed in accordance with a previously reported protocol (Dekkers et al., 2019). Briefly, bulk organoids or MOSs were spun down and rinsed with PBS + 5% BSA, removed from Matrigel by digestion using Cell Recovery Solution (Corning #354253) for 30-60 min, and incubated in 4% PFA at 4 °C for 45 min. The bulk organoids or MOSs were then transferred into a washing buffer (OWB) (0.1% Triton X-100, 2 g BSA per 1 liter of PBS) in a 24-well low-binding plate with 200 µL volume each well and incubated at 4 °C for 15 min. Primary antibodies were then added and incubated overnight at 4°C; the primary antibodies were diluted with OWB in 2X working concentrations, and 200 µL was added per well. On Day 2, primary antibodies were washed off by three rinses in 1 mL of OWB with 2 h intervals in between. Secondary antibodies were added at the third wash at 2X working concentration of 200 µL per well, incubated overnight at 4°C, and washed three times on Day 3 in the same condition as primary antibodies. The nucleus was stained with DAPI, and the organoids or MOSs were cleared using fructose-glycerol clearing solution at room temperature for 20 min. Organoids or MOS (20 µL) in clearing solution were then mounted on a slide and imaged with a Leica SP5 or Leica SP8 confocal microscope. The list of reagents used can be found in the Supplementary table 1.

Gene expression analysis of human colon organoids or MOSs. Human colon organoids or MOSs were collected in the RLT lysis buffer, and the RNA was extracted with the Qiagen RNeasy Mini kit (cat. No. 74104) according to the manufacturer instructions. Total RNA (1 µg) was used to generate complementary DNA (cDNA) with Promega's GoScript Reverse Transcriptase kit (cat. No. A5003) and used to analyze the expression of the following genes: *AXIN2, CCNB1, FABP1, LGR5, MCM2, MUC2, CHGA, EPHB2;* expression levels were normalized to that of *GAPDH*. The list of primers used can be found in the Supplemental table 1.

Generation of Ezrin reporter line. A fluorescent reporter line for Ezrin was established utilizing a strategy described previously (Artegiani et al., 2020). Briefly, cells were transfected with a targeting plasmid containing a tdTomato sequence which is linearized at a defined base position by a specific sgRNA (Supplementary table 1) and Cas9 provided from a second plasmid, encoding mCherry (Schmid-Burgk et al., 2016). These two plasmids are co-transfected with a plasmid encoding the sgRNA specific for the Ezrin C-terminus. All three plasmids were transfected at 5 µg. Transfection was performed using a NEPA21 electroporator and a previously developed protocol (Fujii et al., 2015). Transfected cells were sorted based on mCherry expression. Subsequently, clonal organoids with correct targeting were picked based on Ezrin-tdTomato fluorescence.

Irradiation and chemotherapy. Established PDOs from patients with H&N cancer were passaged to single cell using TrypLE and resuspended in 70% Cultrex reduced growth factor BME type II (R&D systems, cat. No. 3533-010-02). For bulk organoid, the suspension was plated back as per normal passaging, with GF media plus rho-kinase inhibitor (RhkI) (AbMole, cat. No. M1817). MOS were generated using the same single cell/BME suspension as described above, with 10-20 cells calculated per MOS droplet. After two days, PDO organoids were harvested by adding 1 mg/mL of dispase II (Sigma-Aldrich, cat. No. D4693) and incubated for 30 min at 37 ºC to remove BME. Organoids were collected, washed twice with AdDF+++ (Advanced DMEM/F12 containing 1× Glutamax, 10 mM HEPES, and 100/100 U/mL PenStrep) medium, filtered through a 70-mm nylon strainer, and counted. Organoid density was calculated for 25,000 organoids/mL and resuspended in 5% BME/GF medium without RhkI. For MOS, media was refreshed in GF medium to remove RhkI. Either PDO suspension or MOSs (40 µL for each) was dispensed per well using the multidrop combi reagent dispenser into ultra-low attachment 384-well plates (Corning, cat. No. CORN4588). Plates were sealed with Breath-Easy plate seals (Sigma, catalog no. Z380059) and incubated at 37 ºC. Cetuximab (obtained from hospital pharmacy), Gefitinib (Selleckchem, cat. No. S1025), and Afatinib (Selleckchem, cat. No. S1011) were added to MOS using the Tecan D300e digital dispenser. Cetuximab was dissolved in PBS + 0.3% Tween-20, and Afatinib and Gefitinib were dissolved in DMSO. All wells were normalized for the appropriate solvent used and never exceeded 1% for DMSO or 2% for PBS-Tween-20. Drug exposure was performed in triplicate, and irradiation was performed in quadruplicate. Staurosporine (Sigma, cat. No. S5921) was used as a positive control at 1 µM.

Plates were irradiated the day after PDO/MOS dispense by placing each plate in a fixed position on top of a 2-cm polystyrene box and submerging in water at 37 ºC. Plates were irradiated at increasing fractions

of 2 Gray from 2-10 Gray, and a 0 Gray plate was used as the control. Plates were returned to the incubator, and on Day 5, CellTiter-Glo® 3-D Reagent (Promega, cat. No. G9681) was added as per manufacturer's instructions. Luminescence was read out on a Spark multimode microplate reader (Tecan).

For targeted therapy, results were normalized to vehicle (100%) and baseline control (staurosporine, 0%). For irradiation, percent viability was calculated by normalizing each dose of irradiation to the unirradiated (0 Gray). Dose-response curves were plotted using GraphPad Prism software (version 9.0.1)

Histology staining. Organoids or MOSs were harvested from wells and washed twice with AdDF+++ medium to remove residual BME. Organoids or MOS were then fixed with formaldehyde for 24 h and dehydrated in ethanol from 25-70% prior to being embedded in paraffin. Slides were cut at 5-µm thickness. Organoids or MOS were stained with hematoxylin and eosin for H&E staining or with primary antibodies for IHC. Details on primary antibodies for IHC are provided in the Supplemental Table.

Establish and maintenance of human airway organoids from autopsy tissues. The airway organoids were generated as described previously (Sachs et al., 2019). Briefly, the immediate post-mortem specimens were minced with a sterile scalpel into 1-mm^3 fragments in a sterile tissue culture dish. Minced specimens were transferred and incubated in 10 mL digestion media AdDF*(Advanced DMEM/F12 containing 1× Glutamax, 10 mM HEPES, and 100/100 U/mL PenStrep supplemented with 2.5 mg/mL Collagenase D, 0.1 mg/mL DNase I, 10 µM Y-27632 and 100 µg/mL primocin) at 37 ºC for 1–2 h in the orbital shaker. After incubation, remaining fragments were removed by straining through a 70 µm filter. Isolated cells were centrifuged and washed twice with AdDF+++ (Advanced DMEM/F12 containing 1× Glutamax, 10 mM HEPES, and 100/100 U/mL PenStrep). In case of a visible red pellet, erythrocytes were lysed in 2 mL of red blood cell lysis buffer (Roche, cat. No. 11814389001) for 5-8 min at room temperature. Then, 10 mL of AdDF+++ was added, and the cell suspension was centrifuged at 300 x g. Cells were counted, embedded in ice-cold BME, and inoculated in 24-well plates. After at least 15 min at 37 ºC, BME was polymerized. The airway culture media (AdDF+++ supplemented with 500 ng/mL human recombinant R-spondin, 25 ng/mL human recombinant FGF 7, 100 ng/mL human recombinant FGF 10, 100 ng/mL human recombinant Noggin, 500 nM A83-01, 10 µM Y-27632, 500 nM SB202190, 1X B27 supplement, 1.25 mM N-Acetylcysteine, 5 mM Nicotinamide, and 100 µg/mL primocin) was added and refreshed every two to three days. To passage the organoids culture, we removed the airway culture media and mechanically sheared the BME dome with PBS. Then, the mixture of PBS with BME-organoids was collected and centrifuged at 300 x g for 5 min. After centrifugation, the PBS was removed; 2–5 mL TrypLE™ express enzyme was added, and organoids were incubated for 10 min at 37 ºC. Cell suspensions were centrifuged, washed once with PBS, and seeded with BME in a 24-well plate. Airway culture media (Supplemental table 1) was added after BME polymerization.

Infection of human airway MOSs with SARS-CoV-2 and influenza. Human airway MOSs were generated at the density of 20 cells/droplet. After cultured in airway culture media for 3-5 days, the airway MOSs were inoculated with SARS-CoV-2 at a MOI of 2 in airway culture media without Y-27632. The SARS-CoV-2 virus was deposited by the Centers for Disease Control and Prevention and obtained through BEI Resources, NIAID, NIH: SARS-Related Coronavirus 2, Isolate USA-WA1/2020, NR-52281. Biosafety level 3 SARS-CoV-2 studies were performed at the Duke Regional Biocontainment Laboratory, which received partial support from the National Institutes of Health, National Institute of Allergy and Infectious Diseases (UC6-AI058607). Human airway MOSs and virus were incubated for 3 h at 37 ºC. The virus was removed and fresh airway media without Y-27632 was added. Infection proceeded for 48 h. Then, human airway MOSs were washed twice with PBS and collected for downstream analysis. The virus was inactivated following the SOP#308 – method#7 and method#17(Hume et al., 2016). All samples were stored at −80 ºC.

The influenza strain used in this study was an influenza A virus derived from 2009 pandemic swine flu isolate which was engineered to express GFP as previously described (Froggatt et al., 2021). The bulk organoids and MOSs were infected at the MOI of 10. For infection, the MOS droplets were spun down at 200g for 3 min, 200 µL of virus containing buffer (0.4% BSA 1XPBS with Ca^+ and Mg^+) was added to the MOS droplets and followed by an incubation at 37 ºC for 2 h. Then the viral containing supernatants were removed and replaced with the complete media. The efficiency of influenza infection was monitored by fluorescent imaging.

Quantitative RT-qPCR of infected human airway MOS with SARS-CoV-2. Total RNA was extracted using the Direct-zol™ RNA Mini Prep (Zymo) according to the manual. cDNA was synthesized using the High-Capacity cDNA Reverse Transcription Kit (Applied Biosystems™). PCR reactions were prepared using the TaqMan Gene Expression Assay for ACTB (Thermo Fisher) and nCOV_N1 Probe (IDT). RT-qPCR was performed using the Applied Biosystems StepOnePlus™ Real-Time PCR System in a two-step cycling protocol, with a denaturation step at 120 ºC and a combined annealing/extension step at 85 ºC. RT-qPCR measurements represent the average of three independent experiments normalized to β-actin expression. The primers listed in the Supplemental Table were purchased from Integrated DNA Technologies.

Lentivirus production. HEK293T cells were transfected with plasmid encoding either a second-generation anti-HER2 CAR (pHR-SFFV backbone; H3B1) or HER2-mCherry expression (pHR-SFFV backbone) along with pDelta, Vsvg, pAdv viral packaging plasmids at a 15:5:2 ratio using the TransIT-LT1 transfection reagent (cat. No. MIR2300) in a 10 cm cell culture dish. Cells were grown for 48 h after transfection and viral supernatant was harvested and concentrated using LentiX Concentrator (cat. No. 631231). The resultant concentrated virus was 0.45 µm-filtered, aliquoted, snap-frozen, and stored at -80ºC until further use. All lentivirus plasmid constructs were provided as a gift from Wilson Wong.

Generation of HER2$^+$ CRC MOSs and anti-HER2 CAR T-cells. A CRC PDO model was transduced with lentivirus encoding for mCherry-HER2. Briefly, organoid domes were collected and centrifuged at 300 x g for 10 min. They were dissociated to single cells by resuspending the pellet in 1 mL TrypLE Express and incubating for 15 min at 37 ºC. After washing with basal media and centrifugation at 300 x g for 10 min, single cells were resuspended in concentrated lentivirus and incubated for 1 h at 37ºC. Transduced cells were resuspended in BME and plated in 50 µL domes in a 24-well plate. After sufficient time to allow organoid growth and observation of red fluorescence, organoid cells were sorted by flow cytometry and replated in 50 µL BME domes. Sorted mCherry$^+$ organoids were passaged once before use in co-culture experiments. Human PBMCs collected from blood were transduced with lentivirus encoding expression of a second-generation chimeric antigen receptor (CAR) targeted against HER2. In brief, lentivirus concentrate was added to retronectin-coated non-TC-treated 6-well tissue culture plates. Next, the plate was spun at 1200 x g for 90 min at 32ºC. PBMCs that had been activated by ImmunoCult Human CD2/CD3/CD28 activator reagent (cat. No. 10970) for 24 h prior were seeded into each well at 250,000 cells/mL in RPMI-1640 supplemented with 10% FBS and 100U/mL IL-2 (PBMC media). The plate was then spun at 1200 x g for 60 min. Using flow cytometry, we characterized transduction efficiency to be ~43% positive using a low-expression mCherry reporter within the lentiviral construct. The transduced T-cells were cultured at 1 million cells per mL in PBMC media until use in co-culture.

IncuCyte® imaging. Using the IncuCyte® S3 live-cell microscope, we took five images per well every two hours for two days. Quantification of the red fluorescent signal was performed using Incucyte® S3 software with a minimum area of 500 µm^2, to ignore CAR T-cell signal. Red fluorescent signal output was an average of the five images, and post-processing to show fold-change over the time 0 baseline was performed in Microsoft Excel. Plots of the time-series data were generated in JMP.

MOS polarity assay. The line expressing Ezrin-tdTomato were cultured in MOSs (20 cells/droplet) for 3-5 days and the organoid polarities were assessed by the localization of Ezrin-tdTomato. To reverse the polarity of MOSs, the MOSs were spun down at 300g for 5 min in a 15-ml conical tube and resuspended with 10 ml of ice-cold 5 mM EDTA/PBS, followed by incubating the conical tube on a rotating platform at 4 °C for 1 h. The MOS droplets were then pelleted at 300g for 3 min at 4°C and washed one time with ice cold AdDF+++ (Advanced DMEM/F12 containing 1× Glutamax, 10 mM HEPES, and 100/100 U/mL PenStrep) to BME. After aspirating the supernatants, the MOSs were resuspended in the desired complete media and the MOS polarity changes were assessed by confocal or fluorescence imaging. For confocal imaging, the MOSs were plated in a glass bottom imaging plate. 3D imaging of MOSs was performed on a Zeiss LSM 880 confocal microscope using a 10x dry or 20x dry objective. Imaris imaging software (Bitplane) was used for 3D rendering of images.

Glucose vs. fructose tolerance assay. Duodenal intestinal MOSs were generated and seeded in a 96 well glass bottom plate (Greiner Bio-One #655892). On the next day, expansion media was exchanged for

SILAC expansion or EN media with glucose (17.5 mM) or fructose (17.5 mM) by adding 200 μL and then exchanging 150 μL normal expansion media by 150 μL SILAC glucose/fructose 3 times (preparation of media in Supplementary Table). Media was exchanged every 2-4 days. On day 7, 0.2 μL Calcein AM (Biolegend #425201) was added to each well. The MOS were imaged after 30 min incubation using the EVOS FL Auto Imaging System (ThermoFisher). Image analysis was performed using Fiji (protocol Supplementary Table). Data was plotted using GraphPadPrism.

Drug treatments of human airway MOSs infected with SARS-CoV-2. Human airway MOSs were treated with drugs two hours prior to the infection. All samples were then inoculated with SARS-CoV-2 at a MOI of 0.1. After 3 h of incubation, the excess viruses in the media were removed and replaced with the drug (Remdesivir, Camostat, and Chloroquine) containing media for a 48 h additional culture. All the MOS were washed twice with PBS and lysed for RT-qPCR analysis. The virus was inactivated following the SOP#308 – method#7. The drug information is listed in the Supplemental table 1.

Deep learning for organoid/tumorsphere identification. The organoid Detector was trained using the Mask-RCNN (He et al., 2017) implementation in Detectron2 (Kirillov et al., 2020). The configuration used for this study includes a ResNet-50 backbone and an FPN. The training dataset consists of a sample of brightfield images of well-established CRC MOS and paired CAM fluorescence images, all collected by using a Celigo Imaging Cytometer. Ground-truth instance segmentation labels are derived from the fluorescence images by binarizing and identifying each disjoint active region as a separate organoid instance. Since some of the fluorescence images in this training set are not fully saturated, the binarization is performed using a sliding threshold: first, a saturation offset δ is computed as 255 minus the maximum pixel value in the fluorescence image; second, a threshold is computed as $\max(30,90-\delta)$; and finally, each pixel is set to either 255 (if its intensity is larger than this threshold) or 0 (if it is smaller). The network is trained to the resulting labels for 20 epochs, with a learning rate of 0.00025.

An advantage of the Mask-RCNN architecture is that it outputs, for each detected organoid/tumorsphere, a mask indicating which pixels represent part of the organoid/tumorsphere and which do not. When a well is imaged in one or more fluorescence channels as well as brightfield, it is straightforward to measure the fluorescence activity from a given organoid by simply taking a bitwise ‘and’ between this mask and the fluorescence image and summing. However, because the network was trained to predict areas of CAM activity, the mask predicted by the network is biased toward living cells; dead cells are often under-represented in the regions selected by the network. In practice, when a MOS includes both living and dead cells, the dead cells are most frequently found on the outer surface of the organoid/tumorsphere or sprinkled around it, and the network’s mask prediction excludes some fraction of these dead cells. In studies involving the ratio of live-cell stain to dead-cell stain, therefore, the predicted object mask is increased in size to capture all the dead-stain fluorescence signal. The size increase is performed using one iteration of the “dilate” algorithm in OpenCV, with a kernel size of 10x10. Any other organoids/tumorspheres detected by the network which overlap with this expanded region are removed before integrated the fluorescence is computed.

Imaging-based drug assay pipeline. For most drug assays, we generated MOSs at the densities from 20 cells/droplets to 40 cells/droplet. After the initial establishments (2-9 days) in a 24-well non-TC plate, the MOS were automatically dispensed into microwell plates (e.g., 96-well and 384-well plates) using a SpinVessel® coupled with MANTIS® Liquid Handler. The whole-well brightfield images (Day 0 images of the treatment) were acquired by Celigo Imaging Cytometer (Nexcelom Bioscience) after drug dispensing. The whole-well stitched images were exported as tiff files and were segmented automatically using our in-house AI algorithm. The microwell plates were scanned every day to track the growth and morphological changes over the treatment durations. On the day of the CTG assay, the live cell dye, Calcein AM (Thermo Fisher, cat. No. C3100MP), and the dead cell dye, Ethidium Homodimer-2 (Thermo Fisher, cat. No. E3599), were spiked into each well at the working concentrations of 0.5 μM and 2 μM, respectively. After incubation for 45 min at 37 ºC, the stained plates were scanned by Celigo Imaging Cytometer. Images were set to be acquired on brightfield, green, and red fluorescence channels. To avoid overexposure, we set the parameters of gain to 0 and adjusted the exposure time of the green (live cell) fluorescence channel to ensure maximum pixel intensity of the MOSs in untreated wells was below 200. The same strategy was applied for the red (dead cell) fluorescence channel, but we used the positive killing condition wells to guide

the exposure time setup. After scanning, CellTiter-Glo® 3-D Reagent (Promega, catalog no. G9681) reagent was added in a 1:1 ratio with the initial well volume (100 or 40 µL of CTG reagent 96-well plates and 384-well plates, respectively). The plates were placed on a shaker for 20 min at room temperature. Luminescence was then measured by using a plate reader.

For the data analysis, tSA of the segmented objects from each well were measured using the AI algorithm and used for calculating the initial plating variations. The CTG value of each well was then divided by these tSA ratios to yield the adjusted CTG values, which were used to generate viability curves for each drug condition. For the live and dead cell dyes-based imaging assay, the integrated fluorescence intensities of CAM and Eth for each segmented object were calculated. To assess drug response at individual organoid/tumorsphere level, the relative sizes of surface area, the integrated intensities, or the ratios of CAM/EtH were shown on the scatter plot or histogram. The median ratios of the integrated live/dead cell dye intensities from each well were used to plot the dose-dependent drug response.

Supplemental figures:

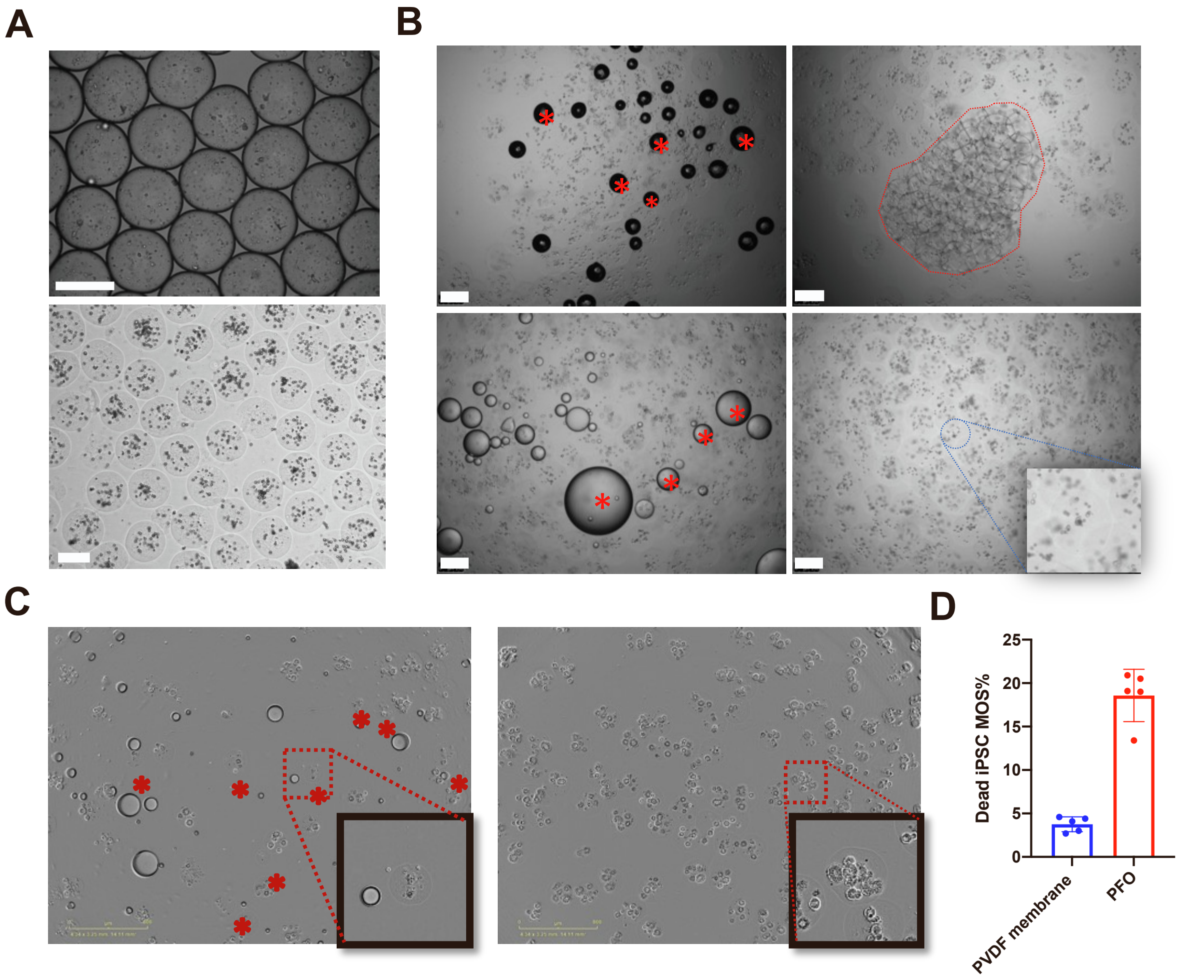

Figure S1. Comparison of demulsification methods. Related to Figure 1. A) A representative image of freshly generated undemulsified droplets (top) and a representative image of MOS droplets after demulsification (bottom); B) A comparison of three different demulsification methods. Top left, undemulsified; top right, antistatic gun; bottom left, PFO; bottom right, PVDF membrane. The red asterisks indicate some of the residue oil droplets after demulsification. The red circle indicates a big clump of unseparated droplets that failed to demulsify. Note the absence of residue oil/surfactant droplets in the bottom right panel image of the MOS droplets after membrane demulsification; C) Left panel, a representative image of iPSC MOSs after the PFO demulsification method. The red asterisks indicate MOSs containing dead iPSCs; the box on the bottom right shows a close-up of one droplet with a cluster of dead iPS cells; right panel, iPSC MOS after PVDF membrane; the box on the bottom right shows a close-up of one droplet with several established, viable colonies of iPSCs. D) The bar graph showing the percentage of dead iPSC MOS using PVDF membrane or PFO method after three days of culture (bars show the mean ± s.d. of 5 random selected views). Scale bars: 250 μm.

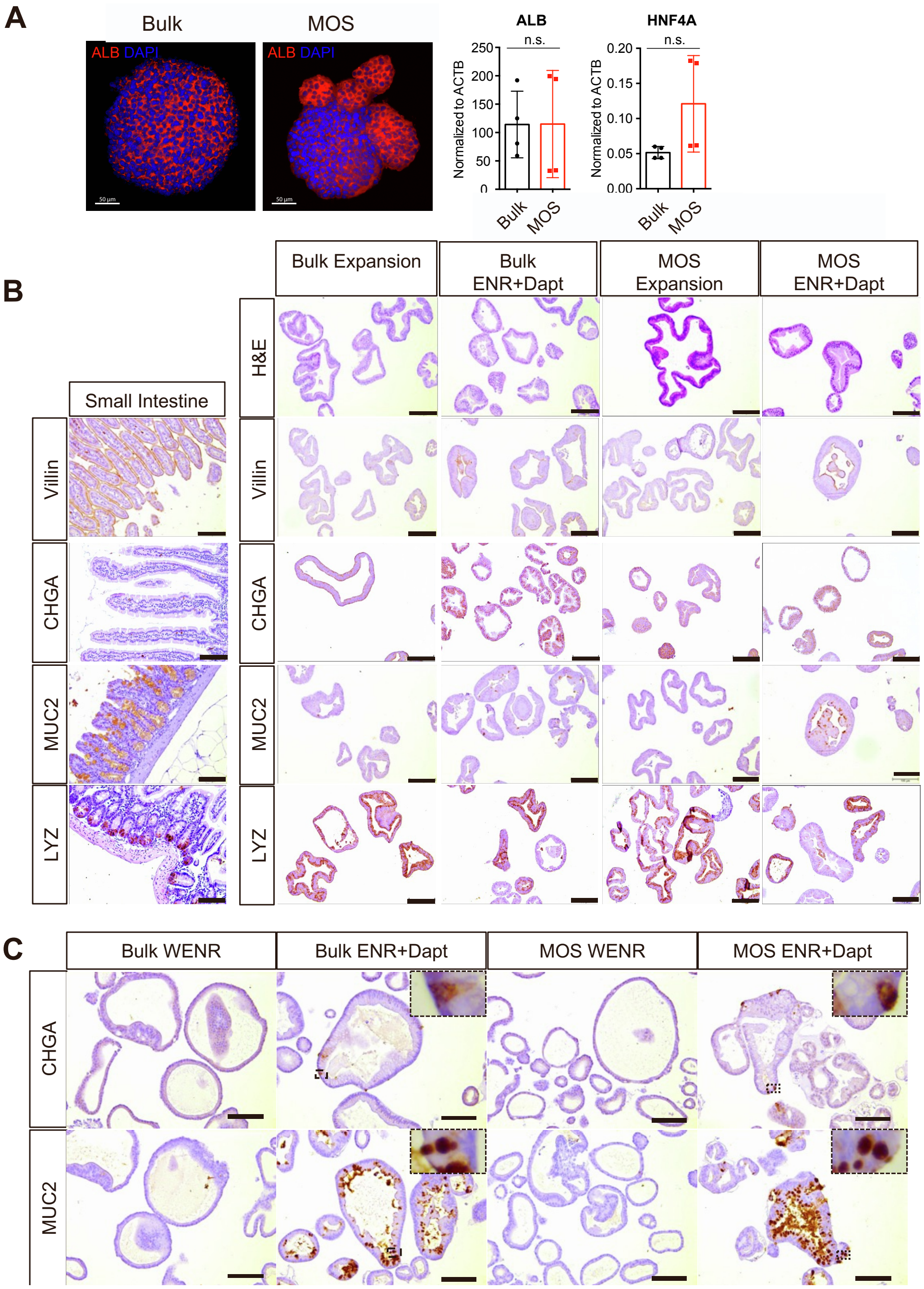

Figure S2. Characterization of human organoids generated as MOS. Related to Figure 1. A) IF staining (left panel) of Albumin of and qPCR analysis (right panel) of Albumin and HNF4A expressen of human fetal liver in both bulk and MOS culture conditions (Bars show the mean ± s.d. of two biological replicates, and each experiment had two technique repeats) (Scale bars: 50 μm); B) Representative stainings of human duodenum organoids in expansion and differentiation medium cultured as Bulk or MOSs. After 5 days of ENR + Dapt culture the organoids showed the typical features of differentiation including the presence of columnar cells and thicker appearence with increased expression of MUC2, VILLIN and CHGA. Mouse small intestine was used as control for the stainings, Scale bars: 100 μm; C) Representative stainings of human organoids derived from small intestine in expansion and differentiation medium cultured as Bulk or MOS. After 5 days of ENR + Dapt culture the organoids showed increased expression of MUC2 (indicating Goblet cells) and CHGA (indicating Neuroendocrine cells). Scale bars: 100 μm.

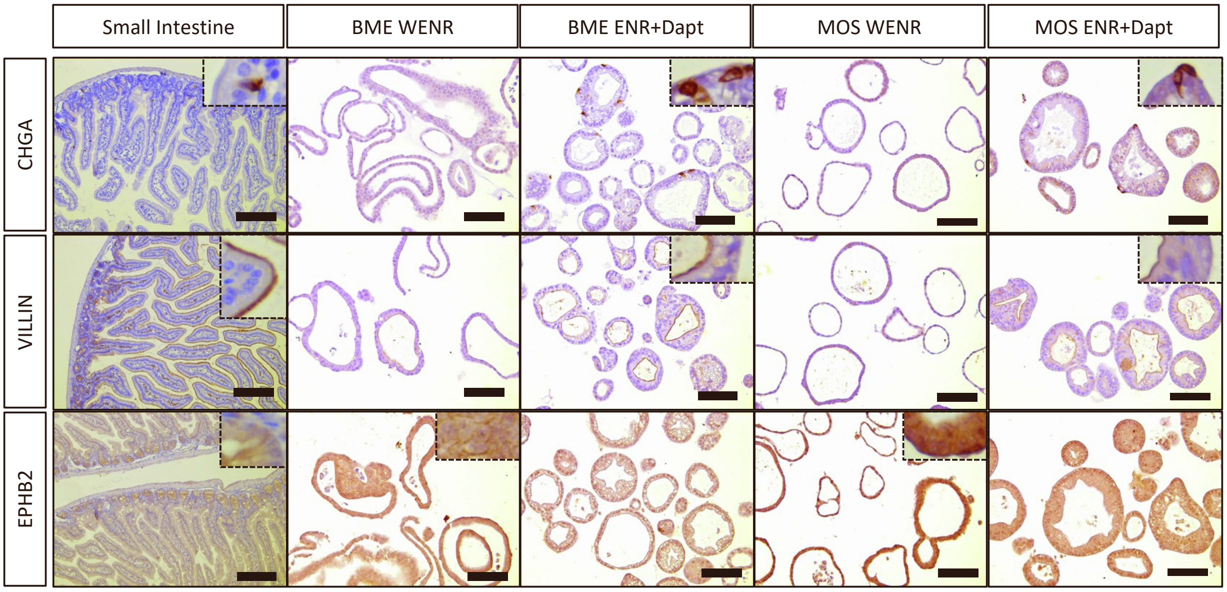

Figure S3. Characterization of MOS differentiation. Related to Figure 2. Representative images of CHGA, VILLIN, and EPHB2 IHC stainings for human colon bulk organoids and MOS cultured in WENR and EN medium showing comparable differentiation. Mouse small intestine was used as control for the staining. Scale bars:100 μm.

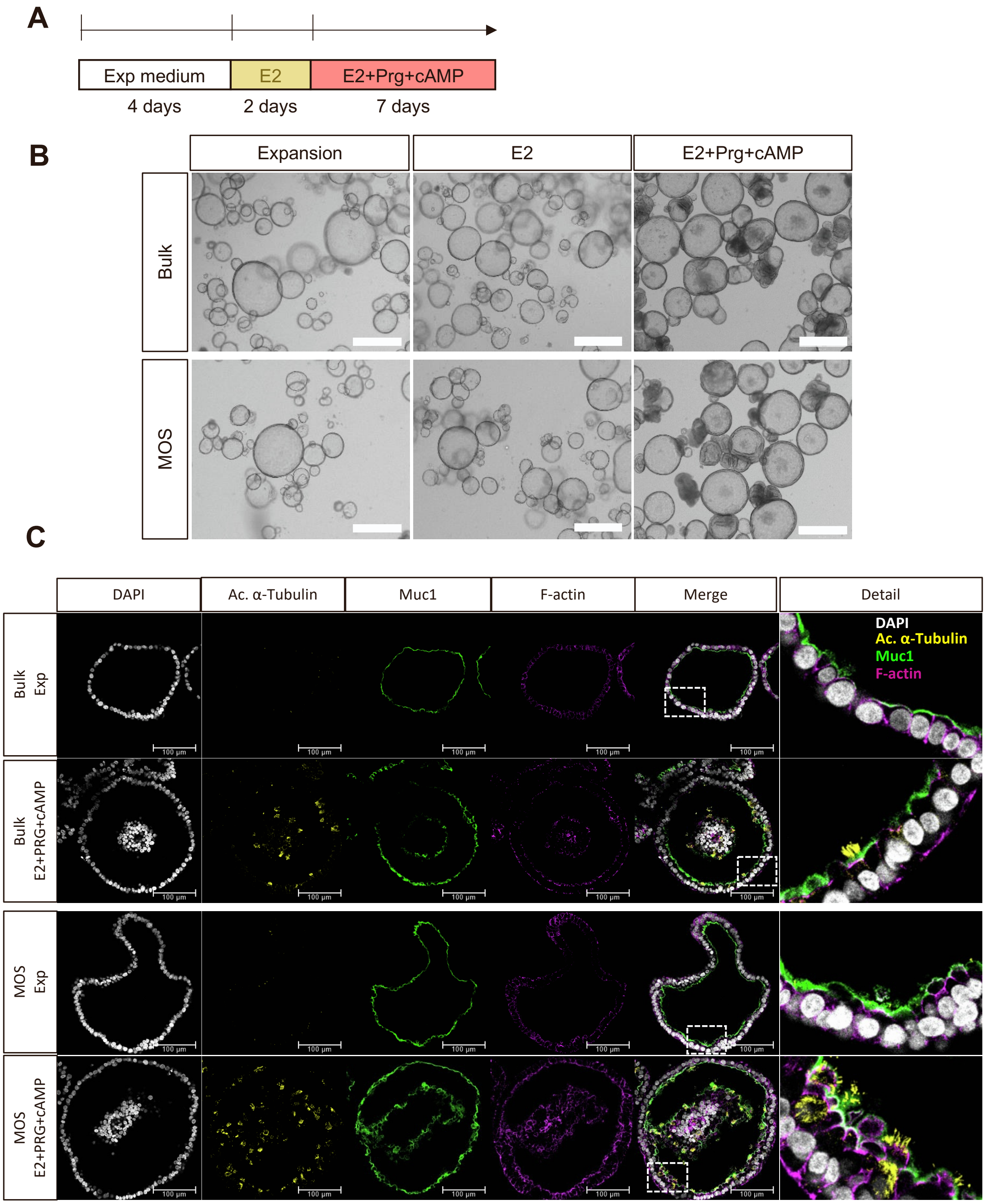

Figure S4. **Characterization of human endometrial MOS. Related to Figure 2**. A) Schematic of the differentiation protocol for human endometrial organoids; B) Representative brightfield pictures of the organoids exposed to differentiation. C) Representative pictures of human endometrial organoids showing differentiation towards ciliated cells (Ac. α-Tubulin) for both bulk and MOS cultures when exposed to progesterone. Scale bars:100 μm.

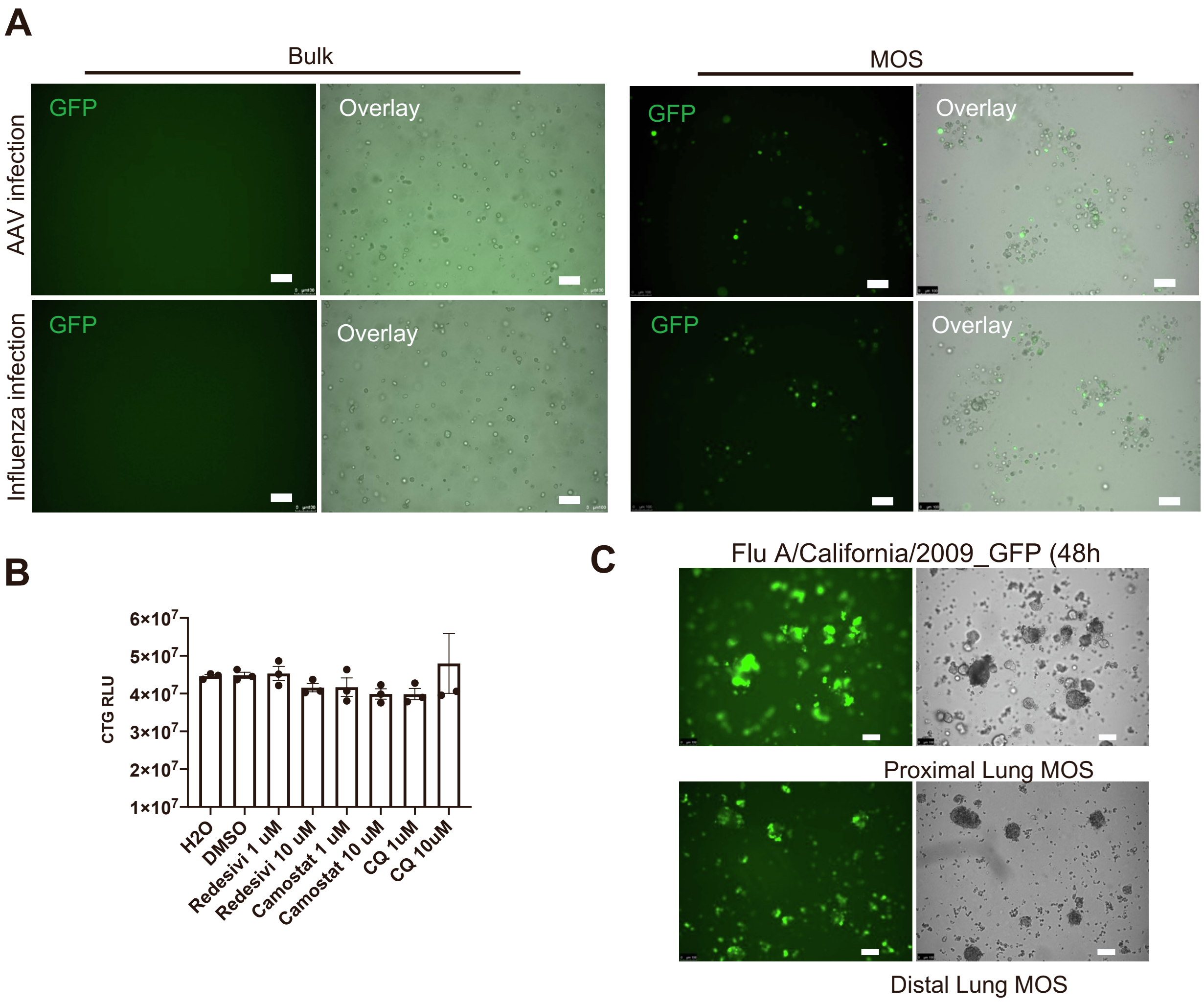

Figure S5. Comparisons of viral infection efficiencies between MOS and bulk organoids. Related to Figure 4. A) Representative images of a CRC bulk organoid and MOS after 36 h of AAV or influenza infections. GFP positive dots indicated successful viral infection (scale bar 100 μm); B) Cell viabilities of distal lung MOS measured by CTG after 48 h treatment with Remdesivir, Camostat, or CQ, respectively (bars show the mean ± s.e.m. of three distinct sample wells); C) Representative images showed proximal and distal lung derived MOS infected with Flu A/California/2009_GFP (scale bars: 100 μm).

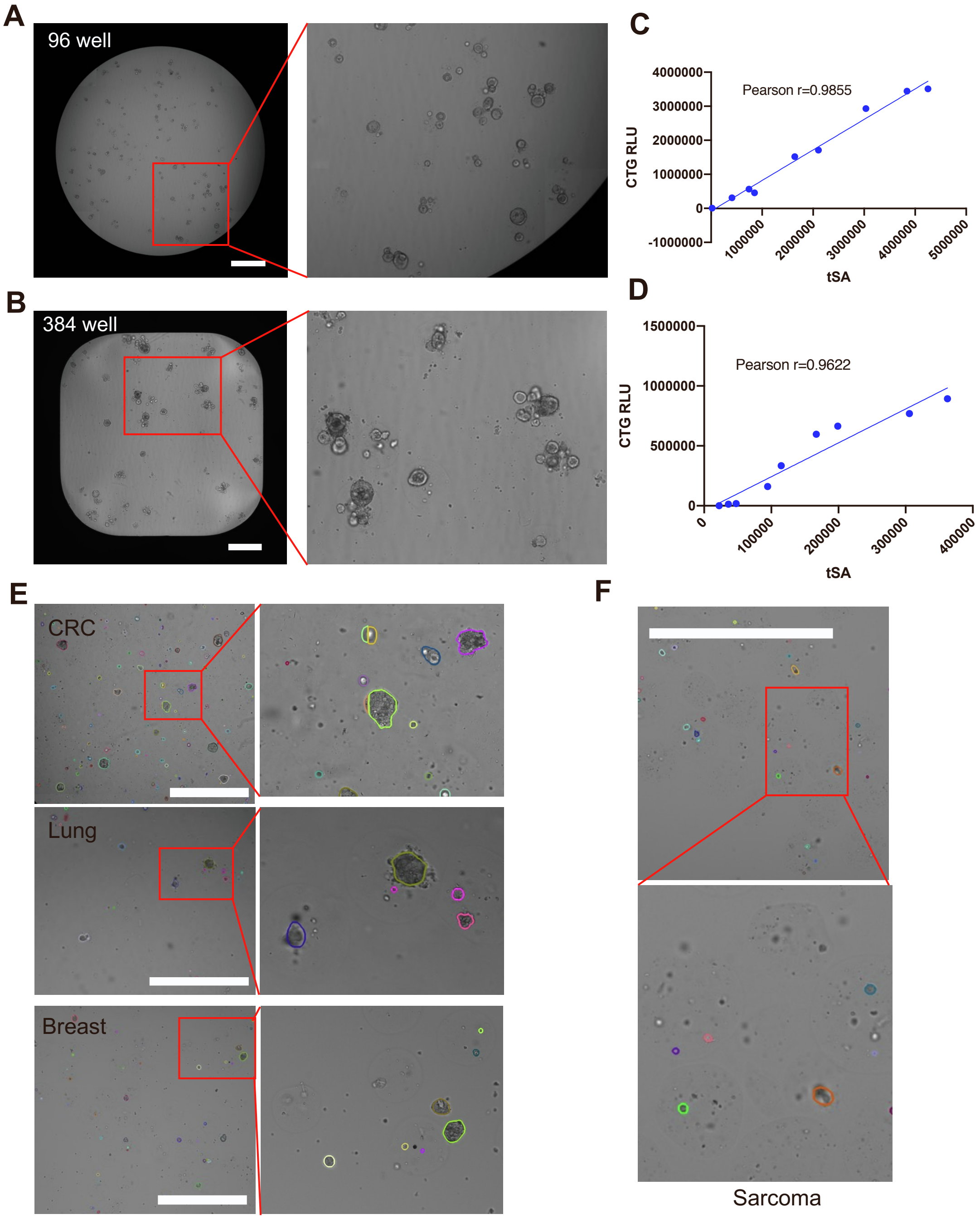

Figure S6. Compatibility of MOS for high throughput imaging. Related to Figures 6 and 7. A) A representative view of the MOS after dispensing into a 96-well plate. Right panel shows a close-up view of several MOS (scale bar 1000 μm); B) A representative view of the MOS after dispensing into a 384-well plate. Right panel shows a close-up view of several MOS (scale bar 1000 μm); C) A liner correlation of CTG raw RLU with the tSA measured by machine learning algorithm in a cystic CRC MOS model; D) A liner correlation of CTG raw RLU with the tSA measured by machine learning algorithm in a dense CRC MOS model. E) Representative images of primary CRC, lung, breast tumor tissue derived MOS with segmentation. Right panel showed the close-up views of the MOS listed on the left panel; F) Representative images of primary sarcoma tissue derived MOS; Scale bars: 1,000 μm.

Supplemental references

Artegiani, B., Hendriks, D., Beumer, J., Kok, R., Zheng, X., Joore, I., Chuva de Sousa Lopes, S., van Zon, J., Tans, S., and Clevers, H. (2020). Fast and efficient generation of knock-in human organoids using homology-independent CRISPR-Cas9 precision genome editing. Nat Cell Biol *22*, 321-331. 10.1038/s41556-020-0472-5.
Dekkers, J.F., Alieva, M., Wellens, L.M., Ariese, H.C.R., Jamieson, P.R., Vonk, A.M., Amatngalim, G.D., Hu, H., Oost, K.C., Snippert, H.J.G., et al. (2019). High-resolution 3D imaging of fixed and cleared organoids. Nat Protoc *14*, 1756-1771. 10.1038/s41596-019-0160-8.
Froggatt, H.M., Harding, A.T., Chaparian, R.R., and Heaton, N.S. (2021). ETV7 limits antiviral gene expression and control of influenza viruses. Sci Signal *14*. 10.1126/scisignal.abe1194.
Fujii, M., Matano, M., Nanki, K., and Sato, T. (2015). Efficient genetic engineering of human intestinal organoids using electroporation. Nature protocols *10*, 1474-1485.
He, K., Gkioxari, G., Dollár, P., and Girshick, R. (2017). Mask r-cnn. pp. 2961-2969.
Hume, A.J., Ames, J., Rennick, L.J., Duprex, W.P., Marzi, A., Tonkiss, J., and Mühlberger, E. (2016). Inactivation of RNA viruses by gamma irradiation: a study on mitigating factors. Viruses *8*, 204.
Kirillov, A., Wu, Y., He, K., and Girshick, R. (2020). Pointrend: Image segmentation as rendering. pp. 9799-9808.
Sachs, N., Papaspyropoulos, A., Zomer-van Ommen, D.D., Heo, I., Bottinger, L., Klay, D., Weeber, F., Huelsz-Prince, G., Iakobachvili, N., Amatngalim, G.D., et al. (2019). Long-term expanding human airway organoids for disease modeling. EMBO J *38*. 10.15252/embj.2018100300.
Schmid-Burgk, J.L., Honing, K., Ebert, T.S., and Hornung, V. (2016). CRISPaint allows modular base-specific gene tagging using a ligase-4-dependent mechanism. Nat Commun *7*, 12338. 10.1038/ncomms12338.
